# Anticancer Potential of Pyridoxine-Based Doxorubicin Derivatives: An In Vitro Study

**DOI:** 10.3390/life14030282

**Published:** 2024-02-20

**Authors:** Rawdah Karwt, Oksana V. Bondar, Mikhail V. Pugachev, Tharaa Mohammad, Aisylu S. Kadyrova, Roman S. Pavelyev, Saleh Alrhmoun, Oleg I. Gnezdilov, Yurii G. Shtyrlin

**Affiliations:** Scientific and Educational Center of Pharmaceutics, Kazan (Volga Region) Federal University, Kazan 420008, Russia; pugachev.mihail@gmail.com (M.V.P.); tharaamohammad1996@gmail.com (T.M.); kaisylu@bk.ru (A.S.K.); rpavelyev@gmail.com (R.S.P.); saleh.alrhmoun1@gmail.com (S.A.); goi@yandex.ru (O.I.G.)

**Keywords:** doxorubicin, anticancer agents, doxorubicin derivatives, pyridoxine, vitamin B6, apoptosis induction, DNA intercalation, cell-cycle arrest, antioxidants

## Abstract

Doxorubicin (DOX) is a prevalent anticancer agent; however, it is unfortunately characterized by high cardiotoxicity, myelosuppression, and multiple other side effects. To overcome DOX limitations, two novel pyridoxine-derived doxorubicin derivatives were synthesized (DOX-1 and DOX-2). In the present study, their antitumor activity and mechanism of action were investigated. Of these two compounds, DOX-2, in which the pyridoxine fragment is attached to the doxorubicin molecule via a C3 linker, revealed higher selectivity against specific cancer cell types compared to doxorubicin and a promising safety profile for conditionally normal cells. However, the compound with a C1 linker (DOX-1) was not characterized by selectivity of antitumor action. It was revealed that DOX-2 obstructs cell cycle progression, induces apoptosis via the mitochondrial pathway without the development of necrosis, and showcases antioxidant capabilities, underlining its cell-regulatory roles. In contrast to doxorubicin’s DNA-centric mechanism, DOX-2 does not interact with nuclear DNA. Given these findings, DOX-2 presents a new promising direction in cancer therapeutics, which is deserving of further in vivo exploration.

## 1. Introduction

Doxorubicin is one of the most potent chemotherapeutic agents isolated from *Streptomyces peucetius* var. *caesius* [1]. It is used widely alone or in combination with other chemotherapeutic agents to treat a wide range of tumors, such as ovarian, lung, breast, prostate, thyroid, gastric, neuroblastoma, and leukemia [2].

Multiple intracellular mechanisms explain the efficacy of DOX. The intercalation into the DNA and Topoisomerase II (Topo II) inhibition are known to be the main mechanisms of DOX activity [3], but other effects are also known. In particular, in combination with dendritic cells, doxorubicin can initiate immunogenic cancer cell death [4]. These events, in addition to its ability to produce adduct formation, are due to the entrance of DOX to the nucleus through the nuclear pores [3]. In the cytoplasm, oxidoreductases convert DOX to the unstable semi-quinone form, which gets converted to DOX again, causing elevated ROS and lipid peroxidation, ultimately causing cell death [5]. DOX also promotes ROS production via other pathways. One of them is by binding to the inner mitochondrial membrane lipids and inhibiting complexes I and II of the respiratory chain [6]. Another is by forming a complex with iron and catalyzing the formation of reactive hydroxyl radicals (OH^−^) [7].

Unfortunately, DOX usage is limited by the severe side effects it can cause. Notably, it has been linked to cardiotoxicity, which can lead to irreversible heart damage in some patients. This has necessitated monitoring of heart function during treatment and often limits the cumulative dose a patient can receive. Moreover, other side effects like bone marrow suppression, nausea, vomiting, and hair loss can also be observed with its administration [8]. More concerns are associated with its hydrophilic nature and short half-life, leading to rapid distribution, excretion, and low bioavailability.

Continuous efforts are also being made in preclinical and clinical settings to discover and validate new drug combinations and formulations that can maximize the therapeutic window of doxorubicin [9]. Chemical modifications of doxorubicin have been actively researched to overcome its limitations. One approach involves doxorubicin linking to targeting moieties or molecules that specifically recognize cancer cells, ensuring that the drug is preferentially delivered to tumor sites [10]. Such targeting moieties can include antibodies, peptides, or other ligands that bind uniquely to receptors overexpressed on cancer cells. Additionally, hybridizing doxorubicin with other therapeutic agents can potentially harness synergistic anticancer effects [11]. This not only enhances the overall therapeutic outcome but might also allow the reduction in doxorubicin’s dose, thereby lessening its adverse effects. The most optimal direction for doxorubicin functionalization is the modification of the molecule at the amino group of the amino-sugar segment. Typically, this approach preserves the molecule’s cytostatic effect [12].

Pyridoxine, commonly known as vitamin B6, has emerged as a potential scaffold for the development of hybrid medicines. The intrinsic bioactivity of pyridoxine, combined with its versatile chemical structure, offers an ideal platform for the conjugation or integration of other therapeutic agents [13]. This scaffold-based approach seeks to exploit the synergy between the inherent biological effects of pyridoxine and the appended therapeutic moieties, such as the synergistic enhancement of the analgesic effect of NSAIDs [14]. Moreover, an active transport system already exists for pyridoxine in living systems [15], which can efficiently increase the transport of pharmacophore groups into living cells and tissues enhancing the drug’s penetration through various biological barriers.

In this research, two doxorubicin derivatives containing pyridoxine fragments were synthesized. The effectiveness, safety, and mechanism of action of these two compounds were further studied on human tumor cells.

## 2. Materials and Methods

### 2.1. Synthesis

Chromatographic purification of compounds was carried out using column chromatography on Acros silica gel (60–200 mesh). The reaction progress and purity of compounds were monitored by TLC on Sorbfil PTLC-AF-A-UF plates. Melting points of the products were determined using a Stanford Research Systems MPA-100 OptiMelt appliance. ^1^H, ^13^C, HSQC, and COSY NMR spectra were recorded on a Bruker Avance 400 WB spectrometer (400.13 and 100.62 MHz). Signals of dimethyl sulfoxide-*d_6_* (δ_H_ 2.50, δ_C_ 39.51) were used as references in the ^1^H and ^13^C NMR spectra. Coupling constants (*J*) are reported in Hz (splitting abbreviations: s, singlet; d, doublet; t, triplet; m, multiplet; br, broad; and combinations thereof). The HPLC/MS experiment was carried out using a TripleTOF 5600 AB Sciex superhigh resolution mass spectrometer (Germany) from the solution in methanol using the turboionic spray (TIS) ionization method with the collision energy with nitrogen molecules of 10 eV.

#### 2.1.1. 2,2,8-trimethyl-4H-[1,3]dioxino[4,5-c]pyridine-5-carboxylic acid (**3**)

KMnO_4_ (4.54 g, 28.7 mmol) was added to a solution of compound **2** (2.00 g, 9.57 mmol) in 50 mL of H_2_O and 50 mL of Me_2_CO. The reaction mixture was stirred at 50 °C for 24 h. Then, the precipitate was filtered off, and the filtrate was concentrated. The resulting solution was acidified with an aqueous solution of hydrochloric acid to pH = 5. The precipitated product was filtered and washed with H_2_O. Yield 71% (1.51 g). White solid, mp 216–217 °C (dec.) (mp 220–221 °C (dec.) [16]); ^1^H NMR (DMSO-*d_6_*, 400 MHz) *δ* 1.49 (s, 6H, C(CH_3_)_2_), 2.35 (s, 3H, CH_3_), 5.09 (s, 2H, CH_2_O), 8.51 (s, 1H, CH_pyr_), 13.34 (br.s, 1H, C(O)OH); ^13^C NMR (DMSO-*d_6_*, 100 MHz) *δ* 18.9 (CH_3pyr_), 24.4 (C(CH_3_)_2_), 59.8 (CH_2_O), 99.5 (C(CH_3_)_2_), 120.7, 128.4, 141.9, 145.6, 151.1, (5 C_pyr_), and 166.5 (C(O)OH).

#### 2.1.2. Ethyl (E)-3-(2,2,8-trimethyl-4H-[1,3]dioxino[4,5-c]pyridin-5-yl)acrylate (**6**)

(2-Ethoxy-2-oxoethyl)triphenylphosphonium chloride (3.72 g, 9.66 mmol) and Et_3_N (4.04 mL, 29.0 mmol) were added sequentially to a solution of compound **5** (2.00 g, 9.66 mmol) in 40 mL of CH_2_Cl_2_. The reaction mixture was stirred for 24 h at 75 °C in an autoclave under pressure. Then, the solvents were evaporated under reduced pressure, and the product was purified by column chromatography (eluent AcOEt). Yield 87% (2.33 g). The ^1^H NMR spectrum is fully described in the literature [17].

#### 2.1.3. (E)-3-(2,2,8-trimethyl-4H-[1,3]dioxino[4,5-c]pyridin-5-yl)acrylic acid (**7**)

A solution of K_2_CO_3_ (0.30 g, 2.17 mmol) in 3 mL of H_2_O was added to a solution of compound **6** (0.60 g, 2.17 mmol) in 30 mL of MeOH. The reaction mixture was stirred at room temperature for 48 h. Then, the solvents were evaporated under reduced pressure at 45 °C. The dry residue was dissolved in H_2_O, and the solution was acidified with an aqueous solution of hydrochloric acid to pH = 5. The precipitated product was filtered and washed with H_2_O. Yield 88% (0.47 g). The ^1^H NMR spectrum is fully described in the literature [17].

#### 2.1.4. N-(3-hydroxy-2-methyl-6-((3,5,12-trihydroxy-3-(2-hydroxyacetyl)-10-methoxy-6,11-dioxo-1,2,3,4,6,11-hexahydrotetracen-1-yl)oxy)tetrahydro-2H-pyran-4-yl)-2,2,8-trimethyl-4H-[1,3]dioxino[4,5-c]pyridine-5-carboxamide (**4**)

HATU (1.70 g, 4.48 mmol) and DIPEA (0.31 mL, 1.79 mmol) were added sequentially to a solution of compound **3** (0.20 g, 0.90 mmol) and doxorubicin (0.52 g, 0.90 mmol) in 30 mL of CH_2_Cl_2_ and DMF (1:1, *v*/*v*). The reaction mixture was stirred for 12 h at room temperature. Then, the solvents were evaporated under reduced pressure at room temperature and the product was purified by column chromatography (eluent CHCl_3_—EtOH, 8:1, *v*/*v*); Yield 43% (0.29 g); dark red solid; mp 165–167 °C (dec.); ^1^H NMR (DMSO-*d_6_*, 400 MHz) *δ* 1.16 (d, 3H, *J* = 6.4, CH_3_), 1.44 (s, 3H, C(CH_3_)_2_), 1.45 (s, 3H, C(CH_3_)_2_), 1.51 (dd, 1H, *J*_1_ = 12.9, *J*_2_ = 4.0, CH_2_), 2.03 (td, 1H, *J*_1_ = 12.9, *J*_2_ = 12.9, *J*_3_ = 3.2, CH_2_), 2.12 (dd, 1H, *J*_1_ = 14.3, *J*_2_ = 5.5, CH_2_), 2.24 (dd, 1H, *J*_1_ = 14.3, *J*_2_ = 1.9, CH_2_), 2.30 (s, 3H, CH_3_), 2.88 (d, 1H, *J* = 18.2, CH_2_), 2.99 (d, 1H, *J* = 18.2, CH_2_), 3.56–3.60 (br.m, 1H, CH), 3.95 (s, 3H, OCH_3_), 4.08–4.20 (br.m, 1H, CH), 4.24 (q, 1H, *J* = 6.4, CH), 4.60 (br.s, 2H, CH_2_OH), 4.82–4.95 (m, 5H, CH_2_OH + CH_2_O + OH + CH), 5.25 (d, 1H, *J* = 3.2, OCHO), 5.44 (s, 1H, OH), 7.53–7.59 (m, 1H, CH_ar_), 7.75–7.87 (m, 2H, 2CH_ar_), 8.17 (d, 1H, *J* = 7.7, NH), 8.20 (s, 1H, CH_pyr_), 13.21 (s, 1H, OH_ar_), 13.98 (s, 1H, OH_ar_); ^13^C NMR (DMSO-*d_6_*, 100 MHz) *δ* 17.1 (CH_3_), 18.6 (CH_3pyr_), 24.4 (C(CH_3_)_2_), 24.5 (C(CH_3_)_2_), 29.3 (CH_2_), 32.0 (CH_2_), 36.4 (CH_2_), 46.0 (CHNH), 56.5 (CH_3_O), 58.9 (CH_2_O), 63.8 (CH_2_OH), 66.6 (CHO), 67.7 (CHO), 69.9 (CHO), 74.9 (C), 99.6 (C(CH_3_)_2_), 100.5 (OCHO), 110.5, 110.6, 118.9, 119.6, 119.8, 125.6, 125.9, 134.0, 134.5, 135.4, 136.1, 138.6, 145.3, 148.5, 154.5, 156.1, 160.7 (12 C_ar_+ 5 C_pyr_), 164.7 (C(O)NH), 186.2 (C(O)), 186.4 (C(O)), and 213.9 (C(O)). HRMS-ESI [M + H]^+^ 749.2551 (calculated for C_38_H_41_N_2_O_14_^+^, 749.2552).

#### 2.1.5. (E)-N-(3-hydroxy-2-methyl-6-((3,5,12-trihydroxy-3-(2-hydroxyacetyl)-10-methoxy-6,11-dioxo-1,2,3,4,6,11-hexahydrotetracen-1-yl)oxy)tetrahydro-2H-pyran-4-yl)-3-(2,2,8-trimethyl-4H-[1,3]dioxino[4,5-c]pyridin-5-yl)acrylamide (**8**)

HATU (0.99 g, 2.61 mmol) and DIPEA (0.18 mL, 1.04 mmol) were added sequentially to a solution of compound **7** (0.13 g, 0.52 mmol) and doxorubicin (0.30 g, 0.52 mmol) in 15 mL of CH_2_Cl_2_ and DMF (2:1, *v*/*v*). The reaction mixture was stirred for 12 h at room temperature. Then, the solvents were evaporated under reduced pressure at room temperature, and the product was purified by column chromatography (eluent CHCl_3_—EtOH, 7:1, *v*/*v*); Yield 56% (0.23 g); dark red solid; mp 175–176 °C (dec.); ^1^H NMR (DMSO-*d_6_*, 400 MHz) *δ* 1.15 (d, 3H, *J* = 6.4, CH_3_), 1.46 (s, 6H, C(CH_3_)_2_), 1.51 (dd, 1H, *J*_1_ = 12.5, *J*_2_ = 4.1, CH_2_), 1.89 (td, 1H, *J*_1_ = 12.5, *J*_2_ = 12.5, *J*_3_ = 2.9, CH_2_), 2.08 (dd, 1H, *J*_1_ = 14.1, *J*_2_ = 5.6, CH_2_), 2.24 (dd, 1H, *J*_1_ = 14.1, *J*_2_ = 2.8, CH_2_), 2.27 (s, 3H, CH_3_), 2.82 (d, 1H, *J* = 18.1, CH_2_), 2.96 (d, 1H, *J* = 18.1, CH_2_), 3.43–3.47 (br.m, 1H, CH), 3.92 (s, 3H, OCH_3_), 4.06–4.16 (br.m, 1H, CH), 4.23 (q, 1H, *J* = 6.4, CH), 4.58 (br.s, 2H, CH_2_OH), 4.80–5.00 (br.m, 5H, CH_2_O + OH + CH_2_OH + CH), 5.24 (d, 1H, *J* = 2.9, OCHO), 5.44 (s, 1H, OH), 6.67 (d, 1H, *J* = 15.9, CH=CH), 7.20 (d, 1H, *J* = 15.9, CH=CH), 7.52–7.54 (m, 1H, CH_ar_), 7.76–7.83 (m, 2H, 2CH_ar_), 7.95 (d, 1H, *J* = 8.3, NH), 8.13 (s, 1H, CH_pyr_), 13.17 (s, 1H, OH_ar_), 13.94 (s, 1H, OH_ar_); ^13^C NMR (DMSO-*d_6_*, 100 MHz) *δ* 17.1 (CH_3_), 18.4 (CH_3pyr_), 24.40 (C(CH_3_)_2_), 24.44 (C(CH_3_)_2_), 29.8 (CH_2_), 32.0 (CH_2_), 36.5 (CH_2_), 45.2 (CHNH), 56.5 (CH_3_O), 58.6 (CH_2_O), 63.8 (CH_2_OH), 66.7 (CHO), 68.1 (CHO), 70.0 (CHO), 74.9 (C), 99.5 (C(CH_3_)_2_), 100.5 (OCHO), 110.5, 110.6, 118.9, 119.6, 119.7, 125.1, 125.4, 125.8, 131.7, 134.0, 134.4, 135.3, 136.1, 138.4, 145.2, 146.8, 154.5, 156.1, 160.7 (CH=CH + 12 C_ar_ + 5 C_pyr_), 163.7 (C(O)NH), 186.1 (C(O)), 186.2 (C(O)), 213.9 (C(O)); HRMS-ESI [M + H]^+^ 775.2702 (calculated for C_40_H_43_N_2_O_14_^+^, 775.2709).

### 2.2. Cell Culture

The study utilized human cell lines including PC-3 (human prostate adenocarcinoma), HSF (primary human skin fibroblasts), MDA-MB-231 (estrogen-negative breast adenocarcinoma), MCF-7 (estrogen-positive breast adenocarcinoma), MSC (multipotent stem cells derived from adipose tissue), SF-539 (brain gliosarcoma), SNB-19 (glioblastoma), A-498 (kidney carcinoma), M-14 (human melanoma), NCI-H322-M (primary bronchioalveolar carcinoma), HCT-15 (colon adenocarcinoma), and HCT-116 (colorectal intestinal carcinoma). We also used immortalized C2C12 mouse myoblasts (ATCC—CRL-1772). Cancer cell lines were obtained from the ATCC collection and generously provided by the Fox Chase Cancer Center (Philadelphia, USA). Conditionally normal cells, including skin fibroblasts and multipotent stem cells, were isolated by our group from postoperative materials (skin and subcutaneous fat) obtained from a conditionally healthy donor. Reagents and consumables for cell culture work were purchased from PanEco (Russia).

Tumor cells were cultured in DMEM supplemented with 10% fetal bovine serum (FBS), 2 mM L-glutamine, 100 μg/mL penicillin, and 100 μg/mL streptomycin. The HSF, MSC, and C2C12 cells were grown in α-MEM with 10% fetal bovine serum, 2 mM L-glutamine, 100 μg/mL penicillin, and 100 μg/mL streptomycin. All cell cultures were maintained under aseptic conditions at 37 °C in a 5% CO_2_ atmosphere. Cells were grown in polystyrene flasks and upon reaching a monolayer, they were detached using a trypsin-EDTA solution (2.5% trypsin, 0.53 mM EDTA) in Dulbecco’s phosphate-buffered saline (DPBS).

### 2.3. Cytotoxicity Studies

The cytotoxicity of the compounds under study was assessed using MTS assay on cell cultures. Cells were seeded into 96-well plates at a concentration of 10,000–20,000 cells/mL. After 24 h, gradient concentrations of the compounds were added to the culture medium and the plates were incubated for 72 h. Subsequently, the culture medium was replaced with MTS reagents (Sigma-Aldrich, Burlington, MA, USA) (MTS:PMS (0.18:0.0092 mg/mL)) and dissolved in phenol-free RPMI 1640 medium. After 2 h of incubation, the resulting water-soluble formazan was measured using the TECAN Infinite M200 Pro microplate reader at wavelengths of 555/750 nm. As a control, equivalent volumes of the solvents in which each compound was dissolved (dH_2_O/DMSO) were used. To calculate the percentage of viable cells relative to the control, which was considered 100%, dose-response curves were plotted against the logarithm of the compound concentration. The concentration of the compound at which cell growth is inhibited by 50% relative to the control was taken as the inhibitory concentration IC50 and calculated using Origin Pro software 8.0.1. The study was conducted in triplicates and in three independent repetitions.

### 2.4. Colony Formation Assay

Cells were cultured in a 24-well plate with 1 mL of DMEM nutrient medium for 24 h. Various concentrations of doxorubicin derivatives were prepared in sterile dH_2_O and added in a volume of 20 microliters to each well containing 980 microliters of fresh nutrient medium the following day. The experiment was conducted in triplicate and in two or three independent repetitions. Only solvent was added to the control wells instead of the test compounds. Incubation with the drugs continued for three days, after which the grown colonies were washed with cold PBS solution and fixed in a fixing buffer (10% acetic acid, 10% methanol in distilled water) for 15 min. The colonies were stained with a 0.4% solution of crystal violet in 20% ethanol for 30 min and then washed 3 times with dH_2_O and analyzed under a microscope. The colonies were then dissolved in 10% acetic acid for 40 min on a shaker, and the absorbance of the lysate was read at 590 nm. Results were expressed as a percentage relative to the control, and a dose–response curve was plotted to determine the IC50 value.

### 2.5. Proliferation Assessment Using an Impedance Biosensor

The effect of the test compounds on the proliferation of multiple cell lines was assessed in real time using the RTCA DP Analyzer (Roche, Basel, Switzerland). For this purpose, we used specialized E-plates containing wells similar in size to a standard 96-well plate, but their bottoms were covered with microelectrodes to assess impedance. When cells adhere to the microelectrodes, the impedance of the well changes, which allows us to calculate the cell index proportional to the cell number. In the experiment, cells were seeded in the E-plate at densities of 20,000 cells per milliliter (180 μL/well). At 24 h post-cell attachment, 20 μL of compounds at various test concentrations or solvent as a control were introduced, and cells were then cultured with real-time impedance registration for 5 days.

### 2.6. Cell Cycle Analysis of Tumor Cells

MCF-7 cells were seeded at a density of 15,000 cells/mL in a 6-well plate in 3 mL of complete medium. After 24 h, the medium was replaced with one containing the studied compounds and incubated for 72 h. After incubation, cells were stained with Hoechst 33342 (Roche, Basel, Switzerland) at a concentration of 6 μg/mL for 30 min in the CO_2_ incubator. The nutrient medium over cells containing the floating cells was collected into tubes, and then the remaining adherent cells were trypsinized and added to the tubes with the floating cells. After this, cells were centrifuged and washed with a DPBS buffer. The fluorescence intensity of cells was measured using the flow cytofluorimeter BD FACSCalibur™ (BD Biosciences, Franklin Lakes, NJ, USA). To identify single cells, direct scatter (FS) and side scatter (SS) measurements were used, and cell doublets were excluded from the analysis using pulse processing based on the area-to-pulse width ratio.

### 2.7. Detection of Apoptosis Induction

MCF-7 cells were seeded at a concentration of 15,000 cells/mL in a 6-well plate. Then, 24 h later the cells were treated with different concentrations of DOX-2 for 72 h. The nutrient medium over cells containing the floating cells was collected into tubes, and then the adherent cells were trypsinized and added to the fraction of floating cells. Then, cells were washed, placed in a binding buffer, stained with Annexin V-FITC/DAPI, and analyzed using the flow cytofluorimeter BD FACSCalibur™ (BD Biosciences, Franklin Lakes, NJ, USA). A total of 1% Triton X-100 was used as a control apoptosis inducer.

### 2.8. Study the Impact on the Mitochondrial Membrane Potential

Cells were seeded in a 12-well plate at a density of 1 × 10^5^ cells/well and incubated for 24 h at 37 °C in a humid atmosphere with 5% CO_2_ prior to treatment. Cells were bathed in complete DMEM media containing the respective compounds, using a solvent (dH_2_O/DMSO) as a negative control. After 72 h, the nutrient medium over cells containing the floating cells was collected into tubes, and then the adherent cells were trypsinized and added to the fraction of floating cells. Cell suspensions were then centrifuged, and the pellet was suspended in phosphate-buffered saline (PBS) to a final density of 5 × 10^5^ cells/mL. Cell suspension in a volume of 250 μL and 500 nM rhodamine 123 were added to a rounded-well plate, followed by incubation for 30 min at 25° C. Measurements were conducted using flow cytometry on the Millipore Guava EasyCyte HT Flow Cytometer System in a green fluorescent channel.

### 2.9. Antioxidant Properties Investigation: Neutralization of the ABTS Radical

Initially, an ABTS solution was prepared in water at a concentration of 7 mM. Subsequently, the radical cation ABTS^•+^ was generated by mixing the original ABTS solution with 2.45 mM ammonium persulfate at a 1:1 ratio. This mixture was then allowed to stand in the dark at room temperature for 12–16 h before use. The diluted ABTS^•+^ solution was prepared in a methanol/water mixture (1:1) reaching an optical density of 0.7 units at 738 nm (~0.7 mM). Next, various concentrations of compounds (ranging from 1000, 500, 250, 125, 62.5, 31.2, 15.6, to 7.8 μM) in a volume of 130 µL in a methanol/water mixture (1:1) were mixed with the ABTS radical cation solution (~0.7 mM) in a volume of 130 µL. The mixture was then incubated at 37 °C in a water bath for 15 min. The optical density was measured at 738 nm using the Infinite M200 PRO (TECAN, Zürich, Switzerland) plate reader. The percentage inhibition of ABTS absorption relative to the control was calculated. A percent inhibition vs. antioxidant concentration graph was plotted and the concentration of the antioxidant that suppresses the optical density of the ABTS radical by 50% (EC50) was determined. The Trolox Equivalent Antioxidant Capacity (TEAC) was also calculated.

### 2.10. Investigation of the Impact on Tubulin Polymerization

To assess the effect of the test compounds on tubulin polymerization, a test kit from Cytoskeleton was used. The plate was pre-warmed to 37 °C for 20 min before starting the assay. Then, 10 µL of the compound of interest, at a 10-fold concentration in an assay buffer, was pipetted into the plate. For comparison, paclitaxel, a polymerization enhancer, and vinblastine, a tubulin polymerization inhibitor, were used. Both effectors were used at a concentration of 10 µM. The assay buffer served as a control. The plate was then incubated at 37 °C for 2 min, after which 100 µL of the diluted tubulin solution (containing 3 mg/mL of tubulin in 80 mM PIPES pH 6.9, 2 mM MgCl_2_, 0.5 mM EGTA, 1 mM GTP, and 7% glycerol) was added. The plate was then immediately placed in the plate reader. Absorbance was measured at 340 nm every minute for 61 readings on a Varioskan LUX (Thermo Scientific, Waltham, MA, USA) plate reader.

### 2.11. Confocal Microscopy

For the analysis of intracellular localization of doxorubicin derivatives, HCT-116 and MCF-7 cells (20,000/mL) were cultured in specific clear-glass bottom cell-imaging 96 well-plates (Eppendorf, Hamburg, Germany) using 10% FBS containing media. A total of 24 h later, the studied compounds DOX-1 and DOX-2 at a concentration of 5 µM were added. Following another 24 h incubation period, Hoechst 33342 (Roche) was added, reaching a final concentration of 1.5 µg/mL. After 15 min, the dye-containing media was removed, and DBBS was added. The plate was subsequently analyzed using confocal microscopy (Zeiss LSM800, Jena, Germany). DOX was administered to the cells two hours before detection at a concentration of 50 µM.

### 2.12. Spectrophotometry

To study the complexation process of the new doxorubicin derivatives DOX-1 and DOX-2 with genomic DNA, the following experiment was conducted. Chicken erythrocyte genomic DNA (Reanal, Budapest, Hungary) solution was prepared by dissolving 6 mg of DNA in 3 mL of Tris-EDTA (TE) buffer and allowed to stay for 2 days at a temperature of +2 °C until clear. The concentration of the initial DNA solution was measured using a nanodrop spectrophotometer (DeNovix, Wilmington, DE, USA). A series of DNA concentrations were prepared in TE buffer (60, 30, 20, 15 μg/mL). Then, DOX, DOX-1, and DOX-2 were added to the DNA samples at a concentration giving 0.7 absorbance and incubated for at least 20 min to allow complex formation. As a control, an equivalent amount of TE buffer in place of the genomic DNA was added to the samples. After incubation, the absorption spectra of DOX, DOX-1, and DOX-2 were recorded on a Perkin Elmer spectrophotometer at wavelengths ranging from 250 to 600 nm. The results were then computed and statistically analyzed using GraphPad Prism software 8.0.1.

### 2.13. Agarose Gel Electrophoresis

To further verify the complexation of DNA and DOX derivatives, electrophoresis of their compositions in agarose gel was performed. Genomic DNA from chicken erythrocytes at a concentration of 30 μg/mL was mixed with DOX (at concentrations ranging from 3.7 to 60 μM) and its derivatives DOX-1 and DOX-2 (at concentrations ranging from 11.2 to 180 μM). The mixtures were then incubated for 30 min. To visualize nucleic acids, the intercalating dye SYBR Green was added at a ratio of 1:10 to the final volume, and the mixtures were incubated in the dark for an additional 15 min. Electrophoretic separation of the nucleic acids and their complexes with DOX, DOX-1, and DOX-2 was carried out in a horizontal chamber in agarose gel (1.4 g of agarose in 200 mL of TAE buffer) under standard conditions at a voltage of 90 volts for 1 h. The electrolyte used was TAE buffer (pH 8.0). The gel was visualized using the Typhoon FLA 9500 gel documentation system on a red channel (532 nm emission) for doxorubicin and a blue channel (473 nm emission) for DNA stained with SYBR Green. 

### 2.14. Comet Assay

PC-3 and MCF-7 cells were seeded into 6-well plates at a concentration of 15,000 cells/mL. After 24 h, DOX, DOX-1, and DOX-2 were added to the medium at concentrations corresponding to their IC25, IC50, and IC70 and incubated for 72 h. Cells were trypsinized and washed, then placed in 0.7% low melting point agarose and mounted on glass slides (200 000 cells/sample). Slides were treated with lysis solution (2.5 M NaCl, 0.1 M disodium EDTA, 1% Triton X-100, 10 mM Tris base, 10% DMSO, pH 10) for 1 h at 4 °C. Next, the samples were placed in an alkaline solution (12 g of NaOH brought to 1 L with water, disodium EDTA 0.2 M, pH >13) and incubated for 30 min at room temperature. Afterward, the slides were washed with electrophoresis 1X TBE buffer (10X TBE: 9.3 g disodium salt EDTA, 108 g Tris base, and 55 g boric acid per 1 L of water) twice and placed in the electrophoresis chamber. Electrophoresis was carried out under neutral conditions in 1X TBE buffer for 30 min at 31 V (at the rate of 1 V/cm). Afterward, the slides were washed in PBS (Ca free, Mg free) 2 times for 5 min, fixed in 96% ethanol for 5 min, and dried. Before analysis, 50 μL of SYBR Green solution (1μL stock + 10 mL TE buffer) was applied to the slides. Using an Observer Z1 fluorescent optical microscope (Carl Zeiss, Jena, Germany), photographs of cells were taken and analyzed in the ImageJ program (Plugin OpenComet).

“Tail moment” (TM) and “Olive moment” (OTM) were calculated using the following formula:

(1) $$TM=\left(\frac{Fluor\;in\;tail}{Fluor\;total}\;\right)\times tail\;lenght$$

(2) OTM=(Tail mean−Head mean)×(Tail DNA %)/100.

### 2.15. Statistical Analysis

The results were calculated and statistically analyzed using GraphPad Prism 9. One-way analysis of variance (ANOVA) was employed for analysis of the results, with differences considered statistically significant at *p* < 0.05. The analyzed variables were presented as the mean ± standard deviation. The significance of differences compared to the control is indicated above the bars, with *p*-values denoted as follows: >0.05 (ns), 0.05–0.01 (*), 0.01–0.001 (**), 0.001–0.0001 (***), <0.0001 (****). Each experiment was repeated at least two-to-three times independently. The figures display either the mean data or data from the most representative experiment.

## 3. Results

### 3.1. Synthesis

**Scheme 1:**
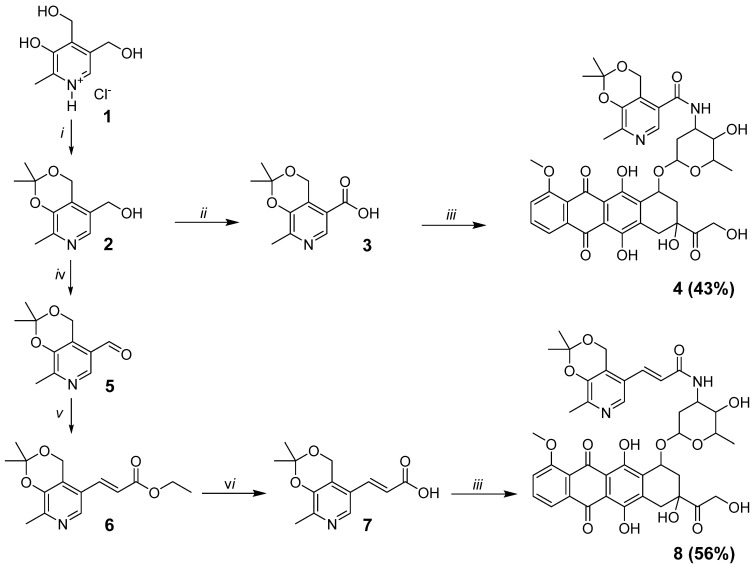
The synthesis of pyridoxine and doxorubicin derivatives linked by a peptide bond. Reagents and reaction conditions: (i) Me_2_CO, HCl; (ii) KMnO_4_, H_2_O/Me_2_CO; (iii) doxorubicin, DIPEA, HATU, DCM; (iv) MnO_2_, CH_2_Cl_2_; (v) (2-Ethoxy-2-oxoethyl)triphenylphosphonium chloride, Et_3_N, DCM; (vi) K_2_CO_3_, MeOH/H_2_O.

The synthesis of pyridoxine and doxorubicin derivatives linked by a peptide bond is shown in Scheme 1.

In the first stage, the pyridoxine-containing derivative of doxorubicin 4 was obtained from pyridoxine in three stages. Initially, the six-membered cyclic ketal of pyridoxine 2 was obtained using a method in the literature [18]. Then, the hydroxymethyl group of the six-membered ketal 2 was oxidized with KMnO_4_ to the corresponding acid 3. The reaction was carried out in an aqueous solution of Me_2_CO under neutral conditions at 50 °C for one day. In the last stage, the final product 4 was obtained with a 43% yield. The reaction was carried out at room temperature for 12 h in a mixture of CH_2_Cl_2_ and DMF (1:1) with the addition of excess HATU and Hunigs base (DIPEA).

In the next stage, a five-step synthesis was used to obtain pyridoxine containing doxorubicin derivative 8 with an acrylamide linker. Carbonyl-containing derivatives 5 were obtained according to a method in the literature [19] by oxidation of the hydroxymethyl group in the fifth position of the six-membered ketal 2 with activated MnO_2_ in CH_2_Cl_2_ at room temperature. Next, alkenyl derivative 6 was synthesized using the Wittig reaction by sequentially adding an equimolar amount of (2-ethoxy-2-oxoethyl)triphenylphosphonium chloride and Et_3_N to the aldehyde 5 solution in CH_2_Cl_2_. The reaction was carried out under pressure at 75 °C for 2 days. Then, carboxylic acid 7 was obtained by hydrolysis under basic conditions of the ester group of derivative 6. The reaction was carried out in MeOH in the presence of a two-fold excess of K_2_CO_3_ at 50 °C for 2 days. In the final stage, a target amide 8 with a yield of 56% was obtained by a similar method, as in the case of compound 4.

**Figure 1:**
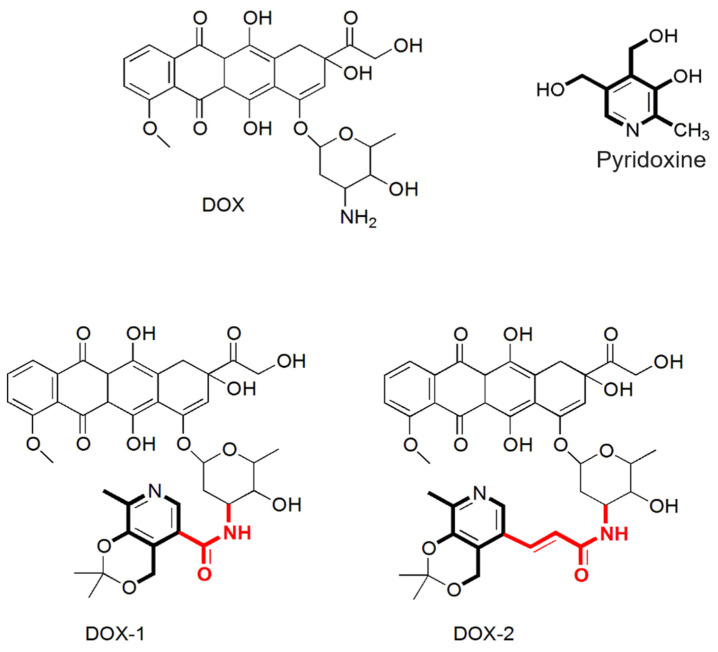
The chemical structure of Doxorubicin (DOX) and its derivatives DOX-1 and DOX-2. Bonds in bold black represent the pyridoxine moiety in the compounds; bonds in bold red represent a linker.

The structures of the synthesized compounds (Figure 1) were confirmed by HSQC, COSY, ^1^H, and ^13^C NMR spectroscopy and mass spectrometry.

### 3.2. Cytotoxicity Studies

#### 3.2.1. MTS Assay

The study of the cytotoxicity of synthesized compounds against tumor cells, as well as against conditionally normal human skin fibroblasts (HSF) and multipotent stem cells (MSC) using MTS assay revealed «structure-activity» patterns (Table 1).

It was demonstrated that unmodified doxorubicin (DOX) exhibited the highest cytotoxic activity. Modification of the doxorubicin structure with pyridoxine fragments according to the previously mentioned scheme resulted in less cytotoxic derivatives: DOX-1 and DOX-2. For instance, DOX-1 and DOX-2 against MCF-7 breast adenocarcinoma cells are 20.9 and 8.5 times less potent than unmodified doxorubicin. For HCT-116 colon carcinoma cells, DOX-1 and DOX-2 derivatives showed 4.2- and 1.3 times reduced cytotoxicity compared to doxorubicin. The selectivity of the DOX-1 derivative is not superior to unmodified doxorubicin. The selectivity index (IC50 HSF/IC50 HCT-116) of DOX and DOX-1 was 1.55 and 1.28, respectively, indicating that they inhibit the proliferation of both tumor and conditionally normal cells similarly. However, the DOX-2 derivative displayed pronounced selectivity against cancer cells compared to conditionally normal HSF and MSC, with the selectivity index for MCF-7 exceeding 260. But for some cancer cell types (HTC-15, NCI-H322M, A-498), DOX-2 appeared to be non-toxic.

By analyzing the dose–response curves (Supplementary Materials), it can be seen that DOX-2 differs in cytotoxic properties from DOX and DOX-1. Increasing the concentration of DOX-2 does not allow complete suppression of cell proliferation. Even in high concentrations, we observe 20–40% of viable cells, which constitute some limit of its efficiency. DOX and DOX-1 can almost completely eliminate cell proliferation at sufficient concentrations.

Keeping in mind the cardiotoxicity of doxorubicin, we additionally evaluated the cytotoxicity of the studied compounds against immortalized mouse myoblasts C2C12, which demonstrated high sensitivity to the studied compounds. Despite the rather low IC50 value (2.8 μM) obtained on these cells, the DOX-2 derivative is 7.26 times less toxic for myoblasts than doxorubicin and 30 times less toxic than DOX-1. It should also be noted that DOX and DOX-1 at sufficiently high concentrations inhibit C2C12 cell proliferation almost completely (by 95% or more), but DOX-2 even at a concentration of 200 μM inhibits C2C12 by no more than 60% (dose–response curves, Supplementary Materials).

By analyzing cells treated with DOX-2 under a microscope, we also noticed a discrepancy between the sufficiently large cell number and the disproportionately low staining. It is likely that DOX-2 inhibits the reduction in the MTS reagent by living cell dehydrogenases to colored formazan. In the case of DOX-2, we could obtain underestimated IC50 results. Given that the MTS tests are sensitive to the metabolic activity of cells and do not always directly reflect their number, the cytotoxicity of the derivatives was also assessed using the colony formation assay and an impedance biosensor, which are based on the direct counting of cell colonies. For cells treated with DOX and DOX-1, no such discrepancy was observed. Based on MTS data, the following test concentrations of DOX were selected for further experiments on MCF-7 cells: 0.01 μM—IC10, 0.02 μM—IC25, 0.05 μM—IC50, 0.2 μM—IC70; and on HCT-116 cells: 0.25 μM—IC25, 1 μM—IC50, 2.5 μM—IC75. The following test concentrations of DOX-1 were selected for further experiments on HCT-116 cells: 1.7 μM—IC25, 4 μM—IC50, and 9 μM—IC75. 

#### 3.2.2. Colony Formation Assay

According to the obtained data (Table 2), the colony formation assay generally agrees with the MTS assay results. The DOX-2 derivative is highly active against MDA-MB231, SNB-19, HCT-116, MCF-7, and PC-3 cells, but non-toxic to HCT-15, NCI-H322M cells, and conditionally normal HSF. However, against immortalized mouse C2C12 myoblasts, DOX-2 was quite toxic with a low IC50 value (0.81 μM); however, if we look at the dose–response curve (Supplementary Materials), we can see that even at a concentration of 100 μM, 30% of C2C12 cells remain viable.

While performing the clonogenic assay, we also noticed that for DOX-2, cell colonies were significantly less stained than expected from visual observations of cell numbers. This could be due to loss of treated cells during multiple washing steps. Apparently, cells treated with DOX-2 become more mobile and detach more easily from the substrate.

#### 3.2.3. Impedance Biosensor Data

In order to reach more accurate real-time cytotoxicity data directly reflecting cell number, DOX-2 was tested on a panel of cell lines using the impedance biosensor RTCA DP Analyzer (Roche, Basel, Switzerland). This device measures the impedance of well-plates containing cells in real time. Viable cells adhere and spread along the bottom of the well, changing its total resistance (impedance). This test does not rely on the metabolic power of cells or any coloring, making it more reliable than conventional cytotoxicity studies.

According to the impedance biosensor (Table 3), the calculated IC50 was considerably higher in comparison with the MTS assay or colony assay, especially for MCF-7 cells (11 and 5 times higher, respectively) and MDA-MB-231 cells (30 and >67 times higher, respectively). A similar pattern was also conserved for PC-3 and SF-539 cells. However, the IC50 of DOX-2 on the HCT-116 cell line was almost identical to the one calculated via the MTS assay. Since the biosensor data directly reflect the cell number and inspire our greatest confidence, the following test concentrations of DOX-2 were selected for further experiments on MCF-7 breast cancer adenocarcinoma cells: 0.4 μM—IC0, 0.7 μM—IC4, 1.5 μM—IC20, 5 μM—IC30, 10 μM—IC60, and 20 μM—IC70.

### 3.3. DOX-2 Impact on the Cell Cycle of Cancer Cells

**Figure 2:**
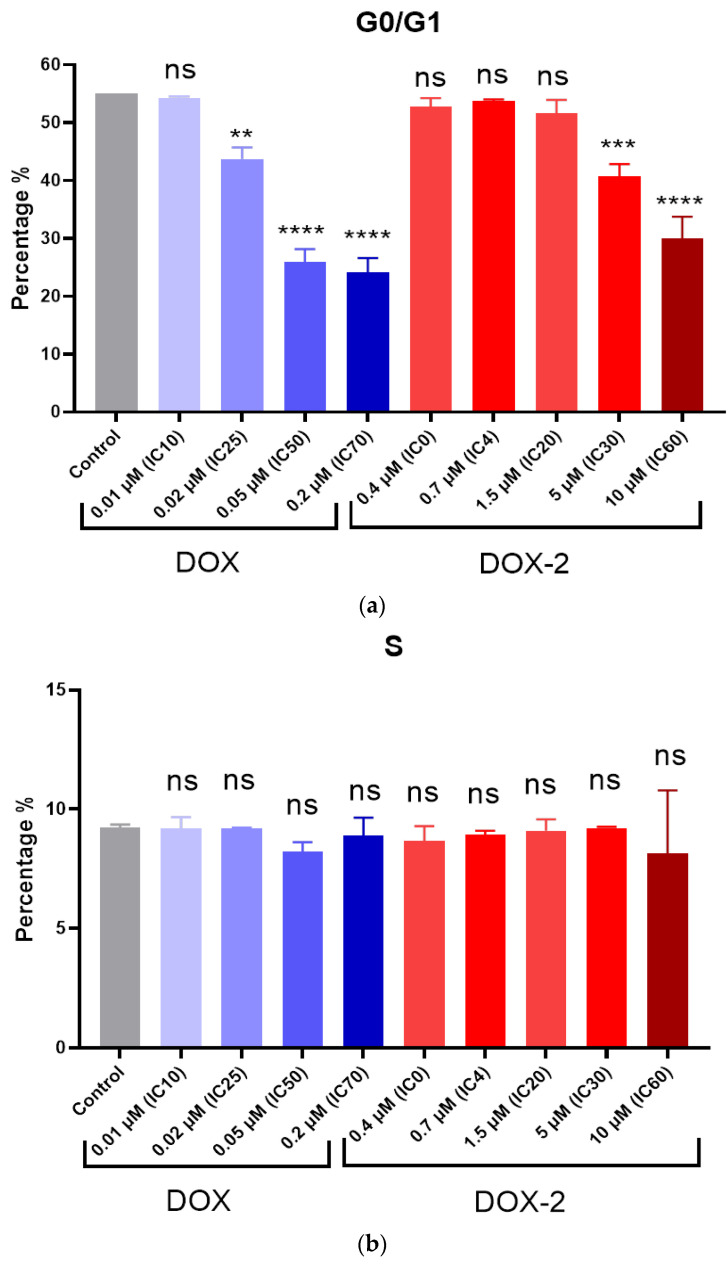

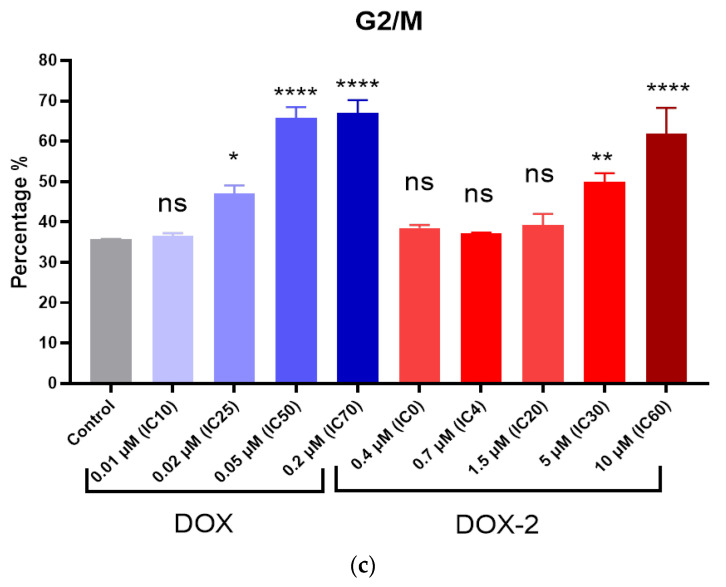
The distribution of MCF-7 tumor cells across cell cycle phases ((**a**) G0/G1 phase, (**b**) S phase, (**c**) G2/M phase) when treated with doxorubicin (blue columns) and its derivatives DOX-2 (red columns) for 72 h using Hoechst 33342 dye as an indicator. *p* > 0.05 (ns), 0.05–0.01 (*), 0.01–0.001 (**), 0.001–0.0001 (***), <0.0001 (****). The experiment was independently repeated three times in duplicate. This figure displays data from the most representative experiment.

Cells treated with various concentrations of DOX and DOX-2 for 72 h were stained with cell-permeable DNA stain Hoechst 33342, of which the fluorescence reflects cellular DNA content and the corresponding phase of the cell cycle. The stained cells were analyzed using flow cytometry. The data obtained on the MCF-7 cell distribution across the cell cycle phases are presented in Figure 2. Both DOX and DOX-2 affected the cell cycle in a similar pattern, decreasing cell percentage in G0/G1 and increasing it in G2/M, which indicates cell-cycle arrest in the G2M phase. The effect was dose dependent and began at concentrations corresponding to IC20–IC30. For doxorubicin, the data obtained correlate well with the literature and also indicate its ability to initiate cell-cycle arrest of tumor cells in the G2/M phase [20,21,22].

### 3.4. Apoptosis Induction in Tumor Cells by Doxorubicin Derivatives

We evaluated apoptosis induction in MCF-7 cells upon treatment with DOX-2 over three days at concentrations corresponding to IC0, IC4, IC20, IC30, and IC60 by measuring Annexin V-FITC and DAPI fluorescence in treated cells. A total of 1% Triton X-100 was used as a control apoptosis inducer. Annexin binds to phosphatidylserine, which is redistributed to the outer membrane of cells in early apoptosis. DAPI stains the nuclei of cells with impaired membrane permeability (late apoptosis and necrosis). 

**Figure 3:**
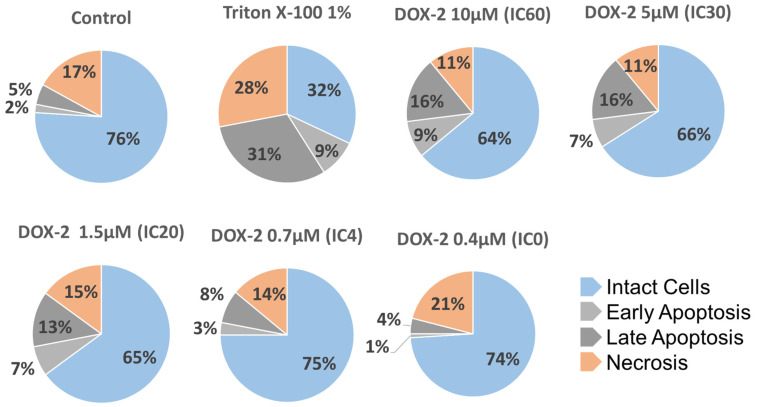
The distribution of MCF-7 cells into apoptosis phases after DOX-2 treatment for 72 h according to Annexin V-FITC/DAPI staining. Treatment with Triton X-100—24 h. The figures present the average data derived from two independent experiments conducted in duplicate.

The results obtained are presented in Figure 3. DOX-2 has been shown to have no significant membrane-damaging activity; at relatively high concentrations corresponding to its IC60, there is no accumulation of necrotic cells. However, when IC20–IC60 concentrations are reached, the percentage of cells in early and late apoptosis increases up to 25%, which indicates moderate apoptotic activity of this compound.

### 3.5. An Impact of Doxorubicin Derivatives on Mitochondrial Membrane Potential

Rhodamine 123 (Rh123) is a cell-permeable cationic green fluorescent dye that is easily sequestered by active mitochondria without cytotoxic effects. It is used to analyze mitochondrial membrane potential [23] and as an indicator of apoptosis via the mitochondrial pathway. Apoptosis induction leads to depolarization of mitochondrial membranes and decreased fluorescence of the indicator.

**Figure 4:**
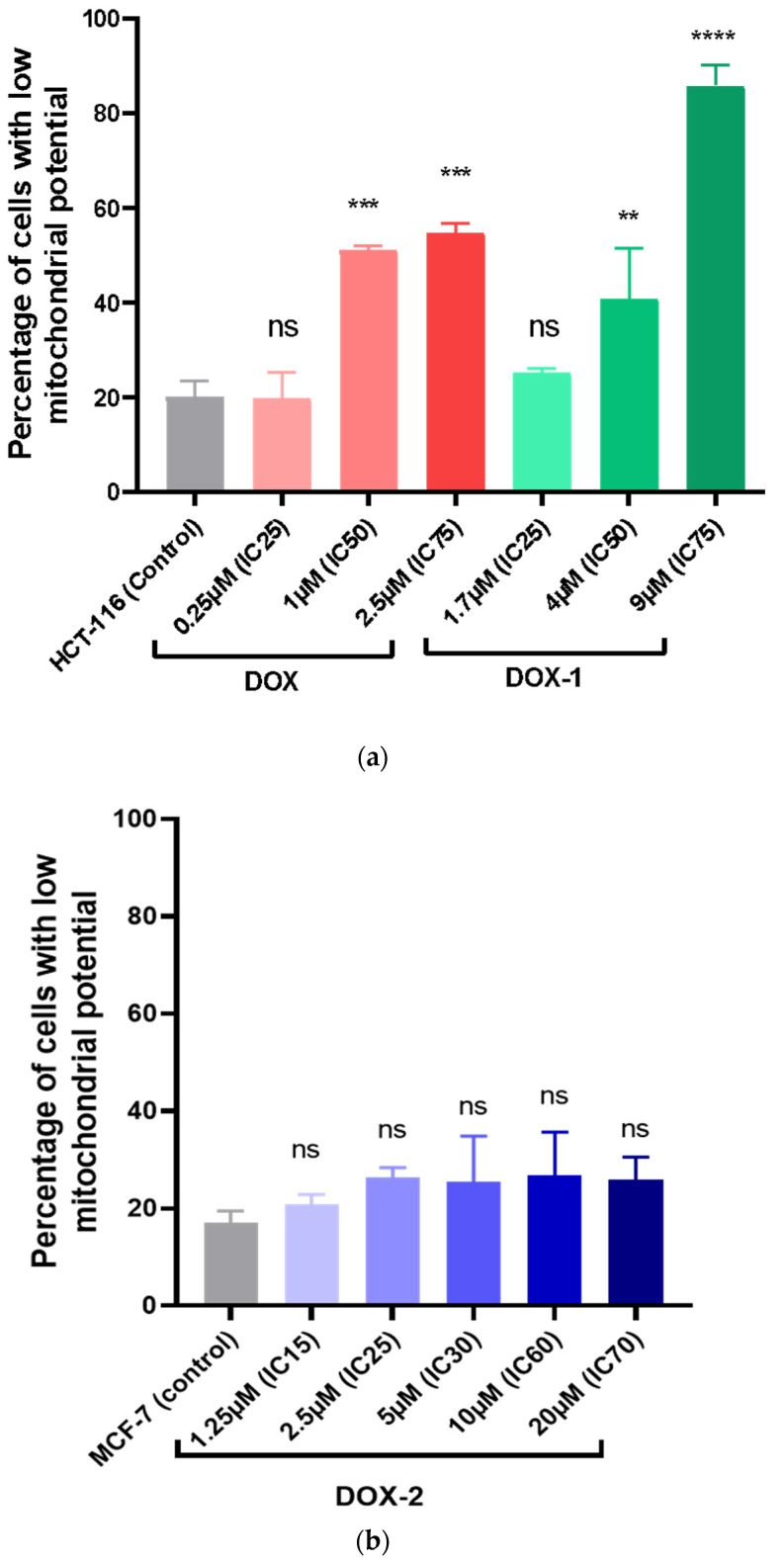
Percentage of cells with reduced mitochondrial potential ΔΨ (cells with low Rh123 fluorescence) when treated with doxorubicin (red columns) and its derivatives (DOX-1 ((**a**), green columns) and DOX-2 ((**b**), blue columns)) for 72 h. *p* > 0.05 (ns), 0.05–0.01 (*), 0.01–0.001 (**), 0.001–0.0001 (***), <0.0001 (****). The figures present the average data derived from two independent experiments conducted in triplicate.

Using flow cytometry, intracellular levels of Rh123 were measured. When cell suspension was stained with Rh123, cells were divided into two subpopulations: cells with high fluorescence (cells with intact mitochondria) and cells with low fluorescence (cells with damaged mitochondria) (Figure 4). 

Treatment of tumor cells with doxorubicin and its derivative DOX-1 at concentrations corresponding to their IC50 or higher is accompanied by the appearance of a significant number of cells with reduced mitochondrial potential (ΔΨ). This indicates the ability of these compounds to induce cell apoptosis via the mitochondrial pathway. 

DOX-2 affects mitochondrial potential more moderately. At a concentration corresponding to IC60, we observed up to 27% of cells with reduced mitochondrial potential. Further increasing the concentration did not result in a greater effect.

### 3.6. Antioxidant Activity of Doxorubicin Derivatives

We assessed the antioxidant activity of doxorubicin derivatives using the ABTS radical neutralization method. Both doxorubicin and its derivative DOX-1 exhibit greater antioxidant potency compared to Trolox by 2.34 and 3.49 times, respectively, positioning their efficacy on par with Quercetin. However, DOX-2 demonstrates lower potency, with an equivalency to Trolox at 0.83. It can be compared in potency to ascorbic acid. (Table 4).

The antioxidant activity of doxorubicin is due to the presence of phenolic hydroxyl groups in its structure surrounded by electron-donating groups and double bonds. The obtained doxorubicin derivatives fully retain phenolic hydroxyl groups in their structures and, therefore, are also antioxidants. However, it is important to note that doxorubicin, and potentially its derivatives when undergoing metabolic transformations within the cell and engaging in redox reactions, tend to function as pro-oxidants, possessing the capability to generate reactive oxygen species (ROS) [24].

### 3.7. Intracellular Distribution of Doxorubicin Derivatives

The obtained pyridoxine-containing doxorubicin derivatives DOX-1 and DOX-2, as well as unmodified doxorubicin, fluoresce in the red region of the spectrum. Therefore, confocal microscopy was used to determine their intracellular distribution. Cells were treated with DOX-1 and DOX-2 at a concentration of 5 μM for 24 h, and DOX at a concentration of 50 μM for 2 h due to its high toxicity and lower fluorescence intensity.

**Figure 5:**
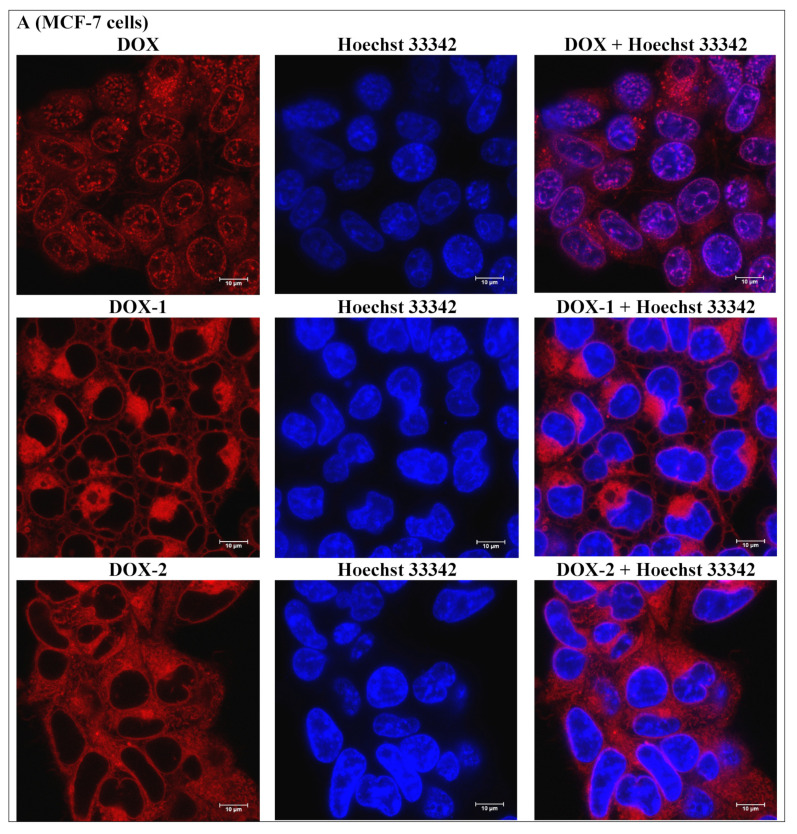

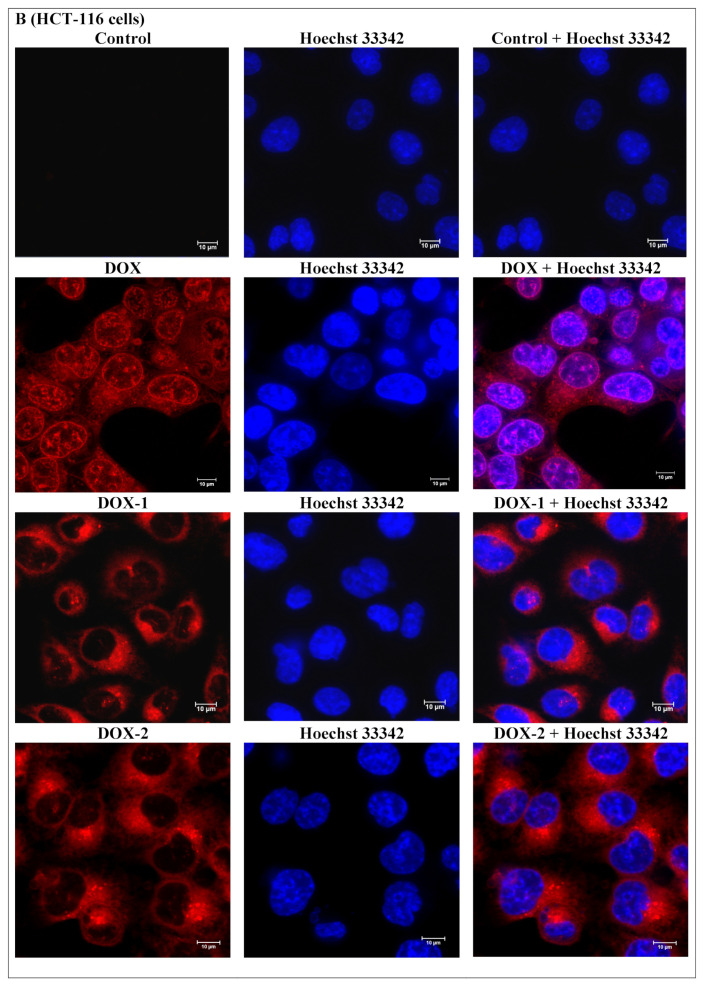
MCF-7 (**A**) and HCT-116 (**B**) tumor cells treated with doxorubicin and its derivatives DOX-1 and DOX-2, as well as with Hoechst 33342 dye for nucleus visualization. DOX-1 and DOX-2—5 μM, 24 h incubation; DOX—50 μM, 2 h incubation. The experiment was independently repeated three times in triplicate. The figures display data from the most representative experiment.

Figure 5 displays micrographs of estrogen-positive breast adenocarcinoma (MCF-7) and colorectal adenocarcinoma cells (HCT-116) treated with DOX, DOX-1, and DOX-2, as well as with the Hoechst 33342 dye for cell nucleus visualization. As observed in the micrographs, DOX-1 and DOX-2 effectively penetrate the barrier of the cytoplasmic membrane and accumulate in the cell’s cytoplasm. However, their further penetration into the cell nuclei is impeded. At the same time, unmodified doxorubicin efficiently permeates and accumulates in the cell nuclei, staining the chromatin.

Therefore, using confocal microscopy, it was revealed that the modified doxorubicin derivatives, DOX-1 and DOX-2, do not penetrate the cell nuclei. The interaction with their primary target of action—DNA molecules—is hampered. This explains their reduced cytotoxic activity against tumor cells compared to doxorubicin.

### 3.8. Evaluation of the Doxorubicin Derivatives Interaction with DNA

#### 3.8.1. Spectrophotometry Assay

Doxorubicin is a DNA intercalator that embeds between the nitrogenous bases of DNA forming doxorubicin-DNA adducts that trigger DNA damage reactions and induce cell death via apoptosis [25]. Doxorubicin forms a covalent bond with guanine on one strand of DNA mediated by formaldehyde and hydrogen bonds with guanine on the opposing strand [25]. Given this, it was of interest to examine whether the new doxorubicin derivatives form complexes with genomic DNA under their interaction in vitro.

**Figure 6:**
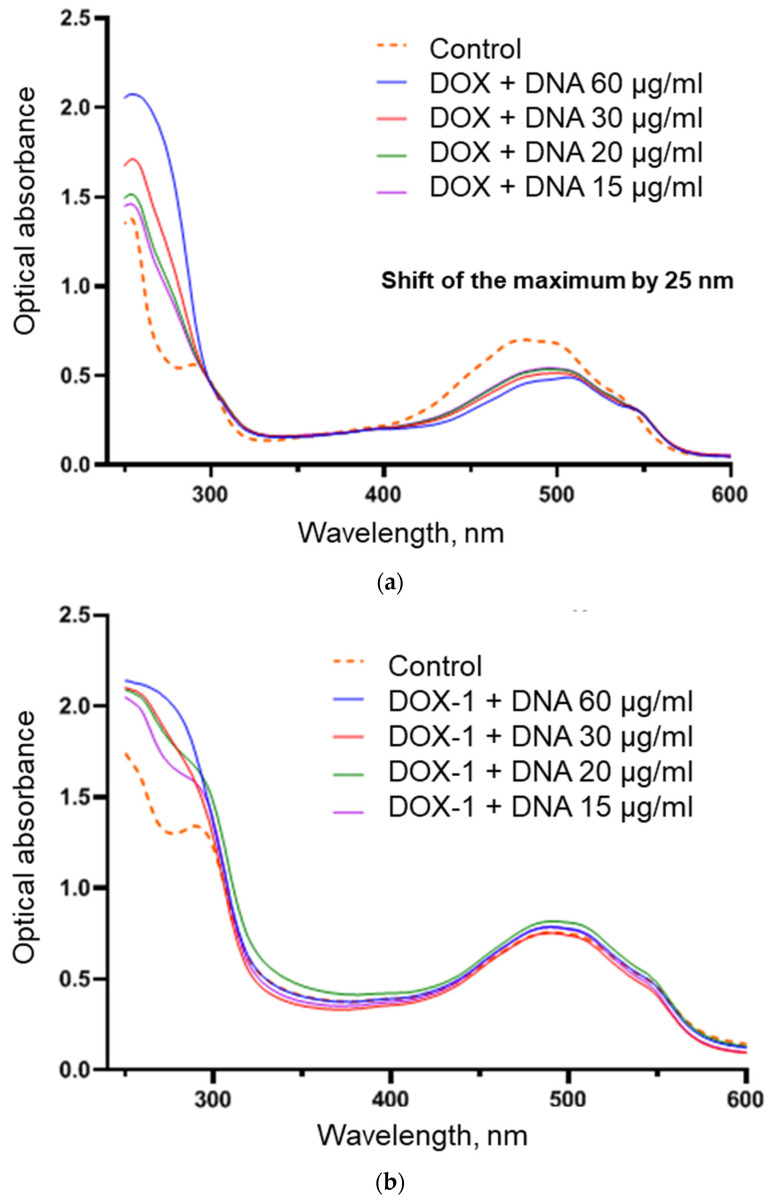

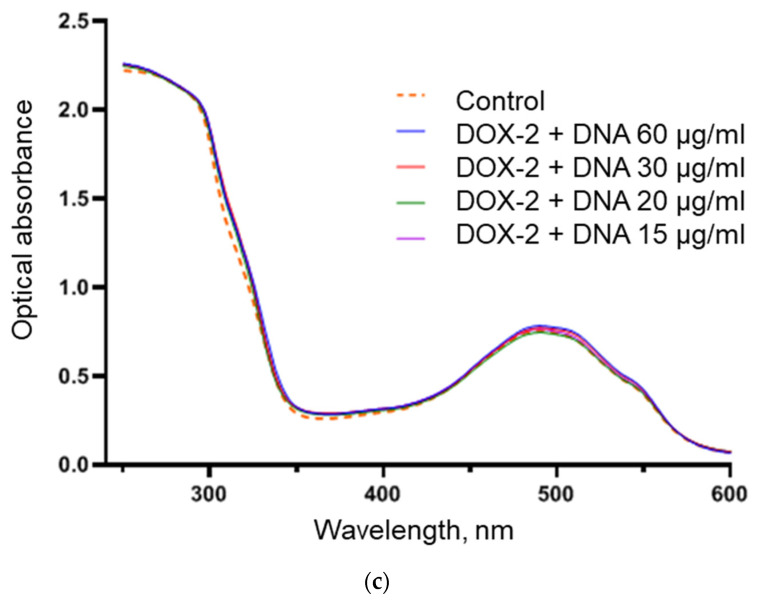
Absorption spectra of doxorubicin (**a**) and its derivatives DOX-1 (**b**) and DOX-2 (**c**) when interacting with chicken erythrocyte genomic DNA. The experiment was independently repeated two times in duplicate. The figures display averaged spectra.

Optical absorption spectra of doxorubicin and its derivatives were recorded after incubating with genomic DNA for 30 min to test complex formation. Doxorubicin has an absorption maximum at 480 nm, while its derivatives DOX-1 and DOX-2 have maxima at 488 nm and 489 nm, respectively. Concentrations of the doxorubicin derivatives were adjusted to yield an optical density of 0.7 at the absorption maximum. The obtained optical spectra of doxorubicin and its derivatives alone and with DNA are shown in Figure 6, and the analysis of the shift in their absorption maxima is presented in Table 5.

When interacting with genomic DNA, significant changes in the doxorubicin optical spectrum were observed. Specifically, its fluorescence peak shifted to a longer wavelength range of 14–25 nm. Additionally, it is demonstrated that, as the DNA concentration increases, the optical absorption of doxorubicin continuously decreases. These findings indicating the intercalation of doxorubicin into DNA are consistent with the literature [26]. Upon interaction with genomic DNA, no significant changes were observed in the optical spectrum of the doxorubicin derivatives DOX-1 and DOX-2, likely indicating a lack of complex formation with DNA.

#### 3.8.2. Gel Electrophoresis

**Figure 7:**
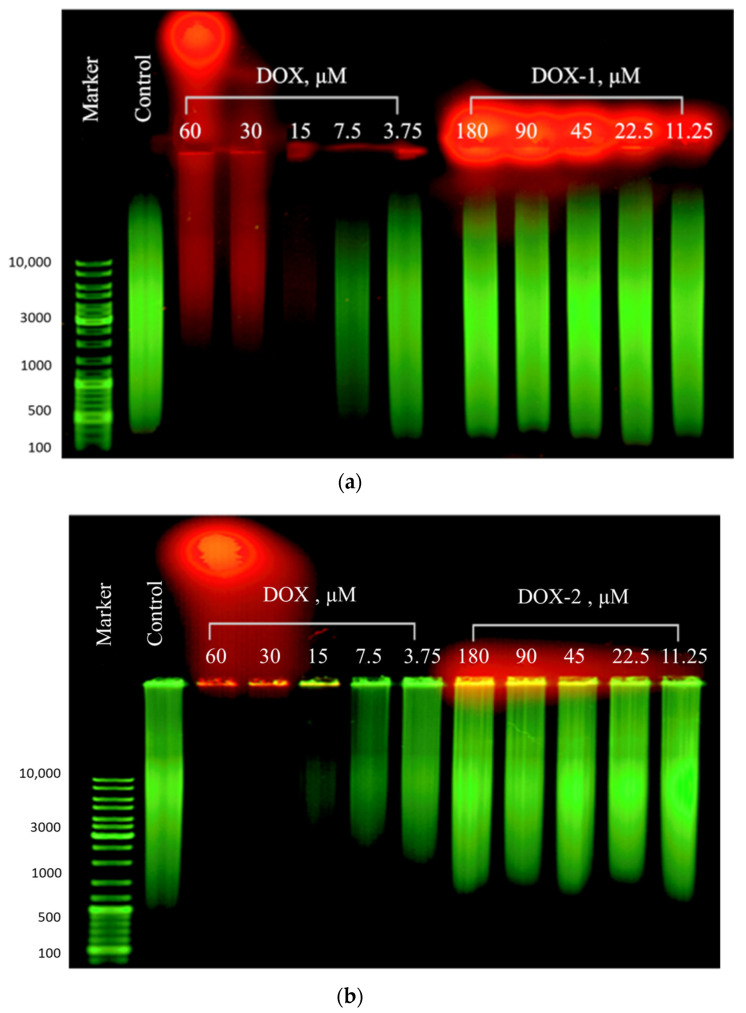
Electrophoregram of genomic DNA compositions with doxorubicin (DOX) and its derivatives DOX-1 (**a**) and DOX-2 (**b**). Red fluorescence corresponds to doxorubicin (emission at 532 nm) and green represents DNA stained with SYBR Green (emission at 473 nm). The experiment was independently repeated two times.

The complex formation with DNA was further investigated using the separation of genomic DNA compositions with doxorubicin derivatives in agarose gel. Before introducing it into the gel, DNA was incubated with varying concentrations of doxorubicin and its derivatives for 15 min, after which the DNA was stained with the intercalating dye—SYBR Green. The obtained electrophoregrams are presented in Figure 7.

As demonstrated in the electrophoregram, the DNA migrates within the gel and gets stained by SYBR Green. When interacting with high concentrations of doxorubicin, the DNA loses its negative charge and does not penetrate the gel. It is shown that 15 μM of doxorubicin is sufficient concentration to completely prevent migration of DNA at a concentration of 30 μg/mL. The studied doxorubicin derivatives DOX-1 and DOX-2 probably do not interact with DNA since DNA migrates freely in the gel and retains its charge upon interaction with these compounds. Hence, we have shown that the doxorubicin derivatives DOX-1 and DOX-2 do not interact with DNA and possess a cytotoxic action mechanism different from intercalation.

### 3.9. DNA-Damaging Activity of Doxorubicin Derivatives

Newly synthesized pyridoxine-containing doxorubicin derivatives do not penetrate into the nuclei. However, we additionally decided to confirm their safety in a DNA Comet assay. The Comet assay is suitable for the detection of DNA strand breaks, crosslinks, and alkali-labile sites induced by a series of physical and chemical agents. DNA migration in an electric field, supposedly proportional to strand breakage, is a proposed estimation of genotoxicity.

In this assay, tumor cells were treated with various concentrations of doxorubicin derivatives for 72 h and reference substances—unmodified doxorubicin and hydrogen peroxide. Treated cells were placed in low melting point agarose and subjected to electrophoresis. 

**Figure 8:**
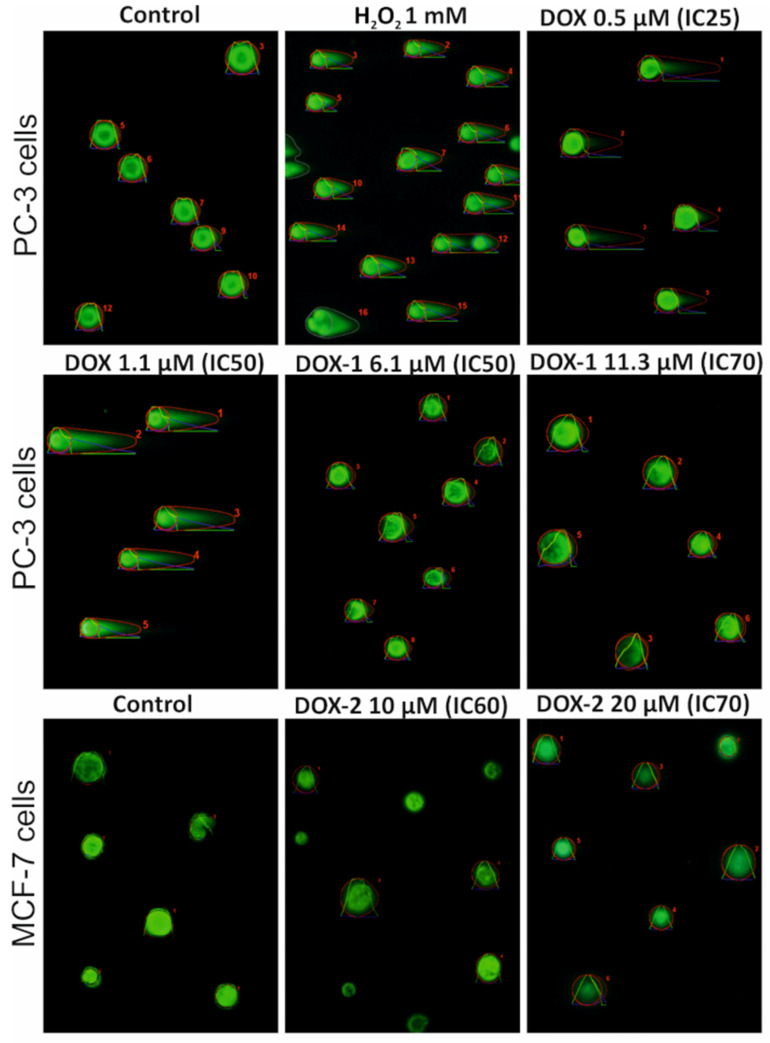
Tumor cells, intact and treated with H_2_O_2_ 1 mM (20 min), DOX (0.5 and 1.1 μM, 72 h), DOX-1 (6.1 and 11.3 μM, 72 h), DOX-2 (10 and 20 μM, 72 h). Agarose gel electrophoresis, SYBR Green I staining. The experiment was independently repeated two times in triplicate.

Intact cells with undamaged DNA are characterized by the absence of comets (Figure 8). Short-term exposure to hydrogen peroxide induces single- and double-strand DNA breaks. Short stretches of DNA leave the cell and appear on the microphotograph in the form of a comet (Figure 8), with a significant increase in the reported parameters of comet tails (Table 6). Doxorubicin, being an intercalator and mutagen, also induces DNA breaks and the appearance of distinct comets, especially at a concentration corresponding to its IC50. Unlike the reference substances, the studied pyridoxine-containing doxorubicin derivatives (DOX-1 and DOX-2) do not cause DNA breaks, and the parameters of comets do not significantly differ from control cells (Figure 8, Table 6).

### 3.10. Effects on Tubulin Polymerization

**Figure 9:**
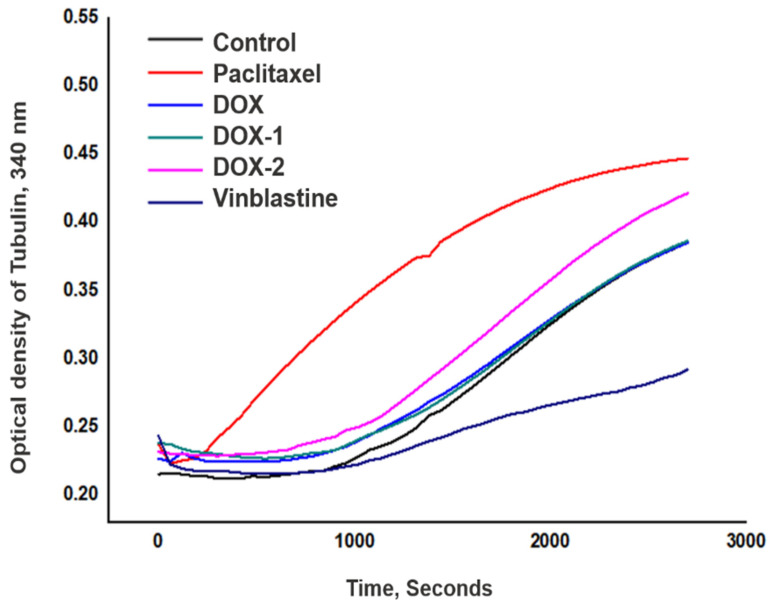
Polymerization kinetics of highly purified porcine neuronal tubulin in the presence of doxorubicin and its derivatives DOX-1 and DOX-2, negative (vinblastine) and positive (paclitaxel) controls. The experiment was independently repeated two times. The figures display data from the most representative experiment.

To reveal the molecular mechanisms of action of the investigated compounds DOX-1 and DOX-2, we evaluated their impact on tubulin polymerization using a test kit from Cytoskeleton. The test is based on the observation that microtubules scatter light proportionally to the concentration of polymerized tubulin. We studied the polymerization of highly purified porcine neuronal tubulin at a concentration of 3 mg/mL at 37 °C in the presence of GTP and 7% glycerol required for polymerization. As positive and negative controls, we used paclitaxel, a polymerization enhancer, and vinblastine, a tubulin polymerization inhibitor. All effectors were used at a concentration of 10 µM, and the resulting curves are shown in Figure 9.

The control polymerization curve presents three phases of microtubule polymerization: nucleation, growth, and a steady-state equilibrium. The maximum initial reaction rate Vmax, calculated from the slope of the curve, was 6.0 ± 1.0 mOD/min. In the presence of paclitaxel, the nucleation phase proceeded faster, with a steeper slope, and Vmax was 8.0, which is 1.2 times higher than in the control reaction. With 10 µM vinblastine, we observed significant inhibition (3-fold) of tubulin polymerization, with Vmax reaching 2.4 mOD/min. The investigated compounds DOX, DOX-1, and DOX-2 had no statistically significant impact on tubulin polymerization; the maximum reaction rate varied within the margin of error (Table 7).

From these findings, we can conclude that, while compounds like paclitaxel and vinblastine can modulate tubulin polymerization, DOX and its derivatives (DOX-1 and DOX-2) have no significant effect on this process, suggesting the presence of a different mechanism of action, influencing other pathways/targets.

## 4. Discussion

Doxorubicin is a widely used anticancer agent known for its potent cytotoxic effects against various cancer types including breast, lung, leukemia, brain, and lymphoma [27]. However, its clinical application is limited by factors such as high cardiotoxicity, several other serious side effects, and a bad pharmacokinetic profile [12]. These limitations highlight the need to improve the selectivity of cancer treatment strategies. The development of hybrid compounds and conjugates [28] is seen as a promising approach to overcome these limitations, offering patients more effective and safer means to combat cancer.

We synthesized two new doxorubicin derivatives containing pyridoxine fragments—DOX-1 and DOX-2—using DIPEA and NATU reagents in three and five steps, respectively. The structures of the synthesized compounds (Figure 2) were confirmed by HSQC, COSY, ^1^H, and ^13^C NMR spectroscopy and mass spectrometry.

The cytotoxicity of new pyridoxine-based doxorubicin derivatives among various cell lines (both normal and cancerous) has been studied using the MTS assay (Table 1). According to the results, DOX-1 is 4.5–26 times less cytotoxic than doxorubicin (except for HCT-15 cells). Both DOX-1 and doxorubicin possess a low selectivity profile, that is, they exhibit the same effect on both tumor and conditionally normal cells. Compound DOX-2 exhibits potent cytotoxic action against almost all cancer cell lines tested, except HCT-15, HCI-H322M, and A-498 cells. The IC50 of DOX-2 exceeds 200 μM for conditionally normal human skin fibroblasts and mesenchymal stem cells. The selectivity index for MCF-7 cells surpasses 260, which is 15 times greater than that of doxorubicin. Of note, DOX-2 was quite cytotoxic against immortalized mouse C2C12 myoblasts with an IC50 of 2.8 μM, but unmodified doxorubicin was 7.3-fold more toxic against myoblasts. For DOX-2-treated cells, we noticed a discrepancy between the formazan formed (a product of metabolic reduction in the MTS reagent by viable cells) and the number of cells observed under the microscope. We hypothesize that DOX-2 may have an inhibitory effect on the MTS reagent reduction by cellular dehydrogenases. For doxorubicin and DOX-1, we did not observe such a discrepancy.

To further validate the anti-proliferative activity of DOX-2, we employed a colony proliferation assay and real-time impedance biosensor (RTCA DP Analyzer (Roche, Basel, Switzerland)). The colony proliferation assay, which measures long-term proliferating capacity and colony formation potential, yielded IC50 values that closely aligned with the values obtained in the MTS assay. However, we also had doubts about the results of the colony test because we noticed that DOX-2 increased cell motility and some cell types were easily detached from the surface during washes. Results obtained with the RTCA-DP impedance biosensor, which directly monitors cell proliferation in real time, presented a different picture. Although the overall safety trend for conventionally normal cells remained unchanged, the IC50 values obtained with RTCA-DP were consistently higher for all cell lines, except HCT-116 cells. This discrepancy highlights the distinct mechanisms and endpoints employed by each assay. The RTCA-DP biosensor measures electrical impedance changes associated with cell adhesion and proliferation and directly reflects the cell number in the well. Given that the impedance biosensor gave us a more realistic picture, we decided to use the IC50 values obtained from this device as a more accurate representation of DOX-2’s anti-proliferative activity in further studies. The following test concentrations were selected for further experiments on MCF-7 breast cancer adenocarcinoma cells: 0.4 μM—IC0, 0.7 μM—IC4, 1.5 μM—IC20, 5 μM—IC30, 10 μM—IC60, 20 μM—IC70. Moving forward, we will explore the underlying mechanisms of DOX-2’s action and investigate its potential as a therapeutic agent for cancer treatment.

Both DOX and DOX-2 exhibited dose-dependent effects on cell cycle progression in MCF-7 cells, with a similar pattern of decreasing cell numbers in the G0/G1 and increasing in the G2/M phase. The observed pattern indicates the presence of a G2/M cell-cycle arrest, which is consistent with the results of other studies of doxorubicin [22]. Probably, cells treated with DOX and DOX-2 do not pass the G2-M DNA damage checkpoint. Cellular DNA receives sufficiently large damage, the repair system fails, and such a cell will not be able to enter mitosis and will undergo apoptosis. Further studies are needed to elucidate the precise mechanisms by which DOX-2 induces cell-cycle arrest.

Further research has shown that DOX-2 increases the percentage of cells in early and late apoptosis up to 25% in MCF-7 breast cancer cells in IC60 according to Annexin/DAPI staining. It is particularly notable that DOX-2 had almost no effect on the appearance of the necrotic cells, indicating its low membrane-damaging activity against the cytoplasmic and nuclear membranes. In the context of cancer therapy, directing tumor cells toward apoptosis is desirable as it ensures their targeted destruction without causing an inflammatory response [29]. 

To elucidate the mechanism of apoptosis induction, the impact of DOX-2 on the mitochondrial membrane potential was investigated. Unlike doxorubicin and DOX-1, which have more pronounced effects on mitochondria, DOX-2 affects mitochondrial potential more moderately. We observed up to 27% of cells with reduced mitochondrial potential in IC60. Mitochondrial potential assessments are in general agreement with Annexin/DAPI staining. Typically, a reduction in the mitochondrial membrane potential is a pivotal event in the intrinsic apoptosis pathway [30], often leading to the release of pro-apoptotic factors and ultimately cell death. 

Using the ABTS radical neutralization test, we confirmed that both doxorubicin and its derivatives DOX-1 and DOX-2 are potent antioxidants. This is not surprising since pyridoxine-modified derivatives retain phenolic hydroxyls in their structures, which are responsible for antioxidant activity. However, it should be remembered that doxorubicin, and potentially its derivatives when undergoing metabolic transformations within the cell and engaging in redox reactions, tend to function as pro-oxidants, possessing the capability to generate reactive oxygen species (ROS) which in turn can trigger apoptosis [24]. Additional studies are needed to conceptualize the prooxidant role of the new doxorubicin derivatives. 

According to confocal microscopy data, DOX-1 and DOX-2 effectively penetrate the cytoplasmic membrane barrier and accumulate in the cytosol, but do not penetrate into nuclei and cannot interact with genomic DNA. Evaluation of optical spectra of new doxorubicin derivatives in the presence of genomic DNA showed that DOX-1 and DOX-2 do not form complexes with DNA and do not belong to intercalators. Electrophoretic separation of genomic DNA compositions with DOX-1 and DOX-2 also confirmed the absence of complexation. DOX-2’s inability to penetrate the nucleus and interact with DNA starkly contrasts with the primary mechanism of action of doxorubicin, which involves DNA intercalation and topoisomerase 2 inhibition [25]. Thus, conjugation with pyridoxine dramatically altered the biological properties of doxorubicin.

The mechanisms of action of DOX-1 and DOX-2 are not only different from doxorubicin but also diverge from other known cytostatic drugs such as vinblastine and paclitaxel [31]. Unlike these two therapeutic compounds, DOX-2 does not impact the tubulin polymerization process. The absence of nuclear penetration by the newly synthesized pyridoxine-containing doxorubicin derivatives and their lack of DNA intercalation offer certain safety advantages. Doxorubicin, a widely used anticancer drug, is known to cause DNA damage and genotoxicity, leading to potential side effects such as cardiomyopathy and secondary malignancies [32]. The comet assay showed that DOX-1 and DOX-2 do not cause DNA fragmentation and are not genotoxic.

The lack of nuclear penetration and DNA damage induction by the pyridoxine-containing doxorubicin derivatives suggests that these compounds may offer a safer alternative to unmodified doxorubicin while retaining pro-apoptotic anti-tumor activity. 

## 5. Conclusions

Herein, we have described novel pyridoxine-based doxorubicin derivatives. Two compounds—DOX-1 and DOX-2—were successfully synthesized and characterized. DOX-1 maintains a similar cytotoxic profile to doxorubicin, showing little distinction in selectivity and efficacy. In contrast, DOX-2 emerges as a notably distinct and promising compound exhibiting high cytotoxicity against specific aggressive cancer cell lines with good selectivity. It induces apoptosis of tumor cells via the mitochondrial pathway, practically without necrosis, which provides a significant therapeutic advantage by ensuring targeted cancer cell elimination with minimal collateral damage. Like doxorubicin, DOX-2 causes cell-cycle arrest in the G2/M phase.

Unlike doxorubicin, which acts mainly through interaction with DNA, new derivatives DOX-1 and DOX-2 do not penetrate into the nucleus and do not interact with DNA. Also, another popular mode of action, inhibition of tubulin polymerization, is not inherent to these compounds.

It was shown that the linker length between the doxorubicin molecule and the pyridoxine fragment influences the selectivity and mechanism of action of the obtained compounds. It was experimentally demonstrated that the compound with C3 linker length (DOX-2) is characterized by low cytotoxicity against conditionally normal cells and high selectivity of antitumor action, while the compound with C1 linker length (DOX-1) has no selectivity.

The characteristics of DOX-2 make it a promising starting point for the development of novel antitumor agents potentially useful in the therapy of aggressive malignancies. Given the attractive properties of the drug, extensive studies in animal models are needed to determine its efficacy and safety profile.

## Figures and Tables

**Table 1:** Cytotoxic concentrations IC50 (μM) of pyridoxine-containing doxorubicin derivatives DOX-1 and DOX-2 against tumor and conditionally normal cells according to MTS assay. The incubation time is three days. Data represent averaging the results of 3–6 independent repetitions of the experiment. Selectivity indices were calculated from the mean IC50 value for HSF cells.

	Cell Line	Compounds		
		DOX	DOX-1	DOX-2
Cancer cells	PC-3	1.05 ± 0.62	5.41 ± 0.88	1.97 ± 0.31
	MDA-MB-231	0.73 ± 0.25	4.05 ± 0.96	1.58 ± 1.03
	HCT-116	1.10 ± 0.64	4.63 ± 0.55	1.43 ± 0.29
	HCT-15	0.46 ± 0.16	>100	>200
	MCF-7	0.09 ± 0.05	1.88 ± 0.48	0.77 ± 0.15
	M14	0.16 ± 0.02	4.29 ± 1.56	2.61 ± 0.24
	NCI-H322M	2.46 ± 1.18	7.20 ± 4.76	>200
	SF-539	0.53 ± 0.35	2.06 ± 0.18	1.74 ± 0.11
	SNB-19	0.10 ± 0.04	5.08 ± 3.40	0.87 ± 0.10
	A-498	0.35 ± 0.06	4.46 ± 3.19	41.7 ± 7.42
Conditionally normal cells	HSF	1.12 ± 0.66	5.86 ± 0.54	>200
	MSC	1.10 ± 0.65	3.87 ± 0.03	>200
	C2C12	0.045 ± 0.004	0.23 ± 0.16	2.80 ± 1.71
Selectivity index	IC50 HSF/IC50 MCF-7	16.45 ± 8.5	3.32 ± 0.87	>260
	IC50 HSF/IC50 HCT-116	1.55 ± 1.45	1.28 ± 0.18	>140
	IC50 HSF/IC50 PC-3	1.72 ± 1.56	1.12 ± 0.24	>100

MCF-7—estrogen-positive breast adenocarcinoma; MDA-MB-231 estrogen-negative breast adenocarcinoma; PC-3—androgen-independent prostate cancer; HCT-116—colon carcinoma; SF-539—brain gliosarcoma; M14—melanoma; SNB-19—glioblastoma; A-498—human kidney carcinoma; NCI-H322M—bronchioalveolar carcinoma; HCT-15—colorectal intestinal carcinoma; HSF—human skin fibroblasts; MSC—human mesenchymal stem cells, adipose-derived; C2C12—immortalized mouse myoblasts.

**Table 2:** Inhibitory concentrations IC50 (μM) of DOX, DOX-1, and DOX-2 against cancer cells and conditionally normal cells by colony formation assay. The incubation time is three days. Data represent averaging the results of 2–3 independent repetitions of the experiment in triplicate.

	Cell Line	Compounds		
		DOX	DOX-1	DOX-2
Cancer cells	PC-3	1.17 ± 0.48	3.20 ± 0.31	1.66 ± 0.82
	HCT-116	0.62 ± 0.14	4.42 ± 3.02	0.91 ± 0.39
	MCF-7	0.06 ± 0.003	0.87 ± 0.12	1.63 ± 0.04
	HCT-15	-	-	109.5 ± 21.9
	MDA-MB-231	-	-	0.34 ± 0.06
	SNB-19	-	-	1.05 ± 0.34
	NCI-H322M	-	-	>100
Conditionally normal cells	HSF	-	-	>100
	C2C12	-	-	0.81 ± 0.07

**Table 3:** IC50 inhibitory concentrations (μM) of DOX-2 against a panel of cell lines as measured by an impedance biosensor (RTCA DP Analyzer (Roche, Basel, Switzerland)). The incubation time is three days. Data represent averaging the results of 2–3 independent repetitions of the experiment in triplicate.

Compound	Cancer Cells					Conditionally Normal Cells
	PC-3	MCF-7	MDA-MB-231	SF-539	HCT-116	HSF
DOX-2	40.26 ± 13.52	8.21 ± 2.93	47.65 ± 26.1	11.61 ± 8.55	1.67 ± 0.02	~100

**Table 4:** Effective EC50 concentrations (μM) of half-maximal ABTS radical inhibition by the tested compounds. Data represent the averaging of three independent experiments conducted in triplicate.

	EC50 (µM)	Trolox Equivalent Antioxidant Capacity (TEAC)
Trolox	15.7 ± 3.4	1.0
Quercetin	3.2 ± 1.6	4.9
Ascorbic acid	15.2 ± 4.8	1.03
DOX	6.7 ± 0.7	2.34
DOX-1	4.5 ± 0.5	3.49
DOX-2	18.9 ± 6.8	0.83

**Table 5:** Changes in the absorption maxima wavelengths of doxorubicin and its derivatives DOX-1 and DOX-2 when interacting with genomic DNA. Data represent the averaging of two independent experiments.

Compound	DNA Concentration (µg/mL)	Absorption Maximum (nm)	Absorption Maximum without DNA (nm)	Shift of the Absorption Maximum Relative to Control (nm)
DOX	60	506	481	25
	30	501		20
	20	495		14
	15	495		14
DOX-1	60	490	492	2
	30	488		4
	20	491		1
	15	490		2
DOX-2	60	491	490	1
	30	490		0
	20	490		0
	15	490		0

**Table 6:** Comet metrics of PC-3 and MCF-7 cells treated with DOX, DOX-1, and DOX-2 for 3 days or with hydrogen peroxide for 20 min. Data were received in the ImageJ program (plugin OpenComet). The experiment was independently repeated two times in triplicate. The table display data from the most representative experiment.

Cell Line	Compounds	Concentration and Corresponding IC	Tail Area	Tail Intensity	Tail Length	Tail DNA %	Tail Moment	Olive Moment
PC-3	Control	-	928	37	13	7.9	1.59	2.64
	H_2_O_2_	100 μM	2516	19	41	9.2	6.29	5.26
		1000 μM	1096	38	30	24.2	9.60	6.85
	DOX	0.5 µM (IC25)	2215	66	51	22.7	13.45	8.13
		1.1 µM (IC50)	1902	74	51	35.2	24.57	11.64
	DOX-1	3 µM (IC25)	1094	34	10	6.7	1.07	2.48
		6.1 µM (IC50)	1196	30	17	6.7	1.36	2.24
		11.3 µM (IC70)	1403	37	12	11.4	1.87	3.79
MCF-7	Control	-	1880	67	6	7.2	0.47	2.80
	DOX-2	5 µM (IC30)	1254	10	4.9	1.3	0.14	0.73
		10 µM (IC60)	637	21	1.5	5.1	0.21	1.42
		20 µM (IC70)	876	20	5.8	1.9	0.25	1.01

**Table 7:** Maximum initial rate of tubulin polymerization in the presence of test compounds (DOX, DOX-1, and DOX-2), positive (paclitaxel), and negative (vinblastine) controls. * *p* < 0.5. The data are an average of two independent repetitions.

	Maximum Initial Rate of Polymerization V Max (mOD/Min)
Control	6.7
Paclitaxel	8 *
Vinblastine	2.4 *
DOX	5.5
DOX-1	5.5
DOX-2	6.8

## Data Availability

The data presented in this study are available upon request from the corresponding authors.

## Supplementary Materials

The following supporting information can be downloaded at: https://www.mdpi.com/article/10.3390/life14030282/s1, Figure S1: Appendix to Table 1. Sigmoidal dose-response relationships used to determine IC50 values of the studied compounds in the MTS assay; Figure S2: Appendix to Table 2. Sigmoidal dose-response relationships used to determine IC50 values of the studied compounds in the colony formation assay.
