# Supplementary material for: Anticancer Potential of Pyridoxine-Based Doxorubicin Derivatives: An In Vitro Study
Source: Life (Basel). 2024 Feb 20;14(3):282. doi: 10.3390/life14030282 (PMC10970924; doi:10.3390/life14030282)
Supplement: Supplementary file 1 [file life-14-00282-s001.zip › life-2801565-supplementary.pdf]

Supplementary Materials

Figure S1. Appendix to Table 1. Sigmoidal dose-response relationships used to determine IC50 values of the studied compounds in the MTS assay. Data are averages of 2-3 independent repeats, mean ± SD.

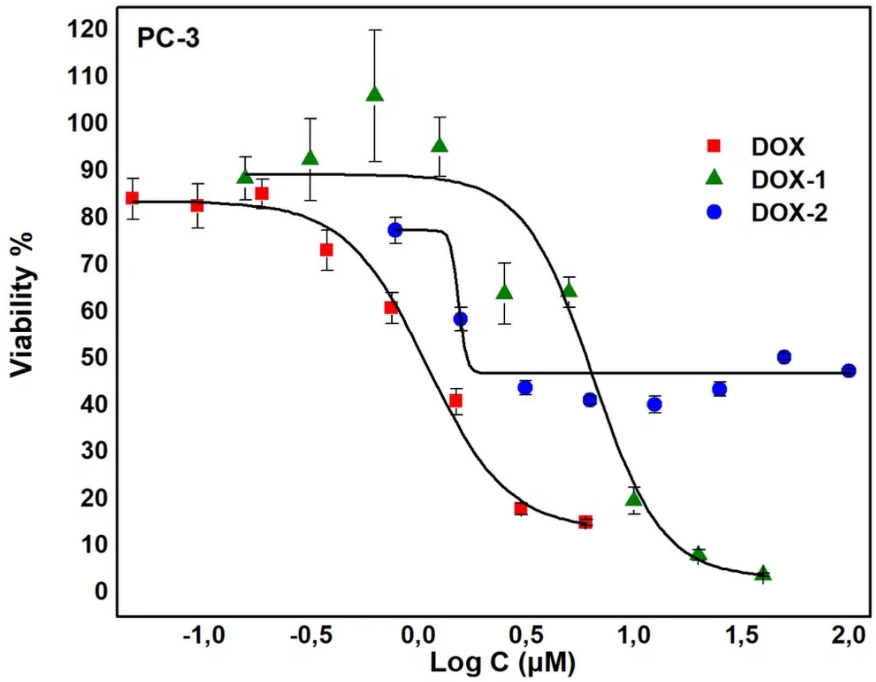

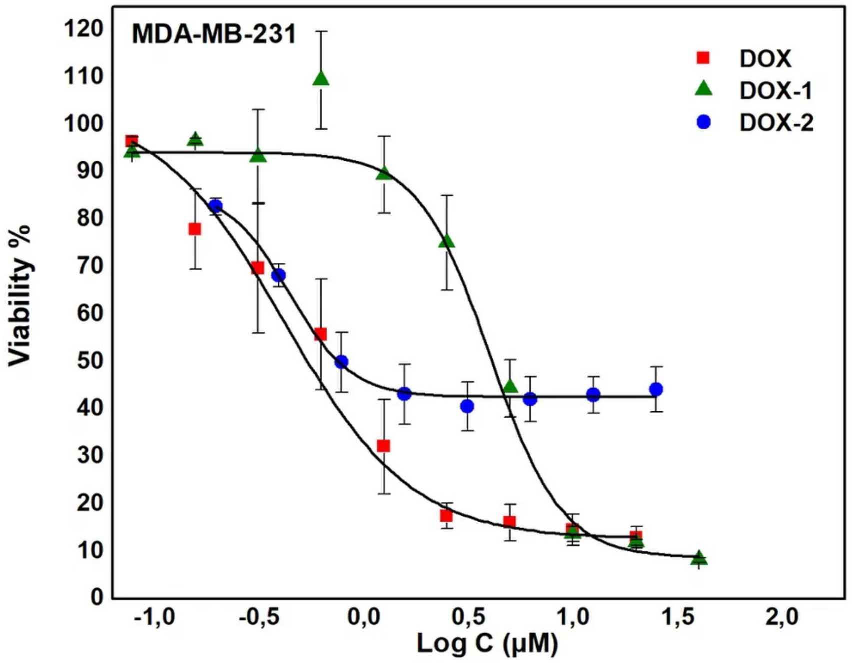

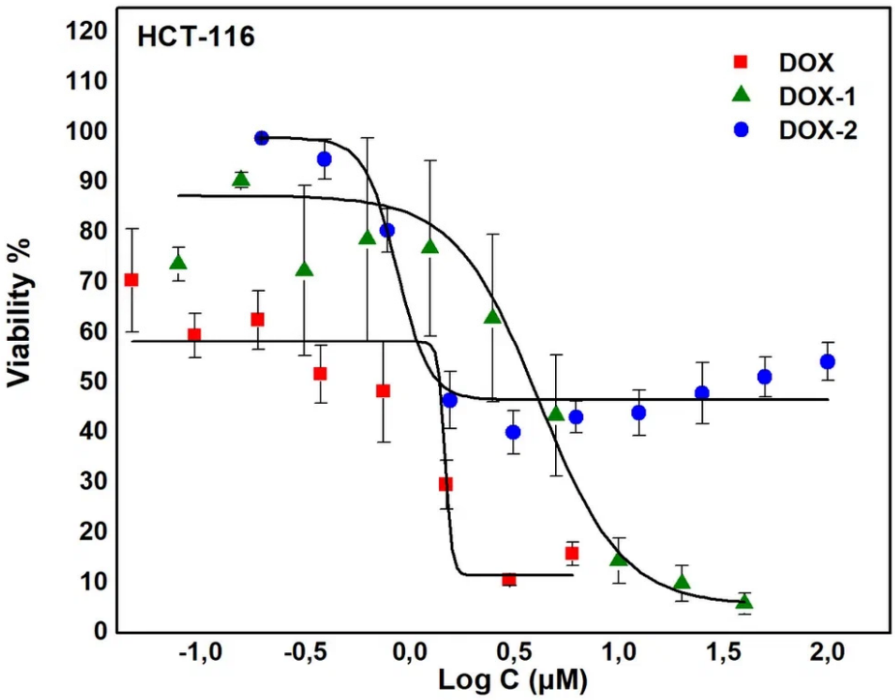
HCT-116
DOX
DOX-1
DOX-2
Viability %
Log C (µM)

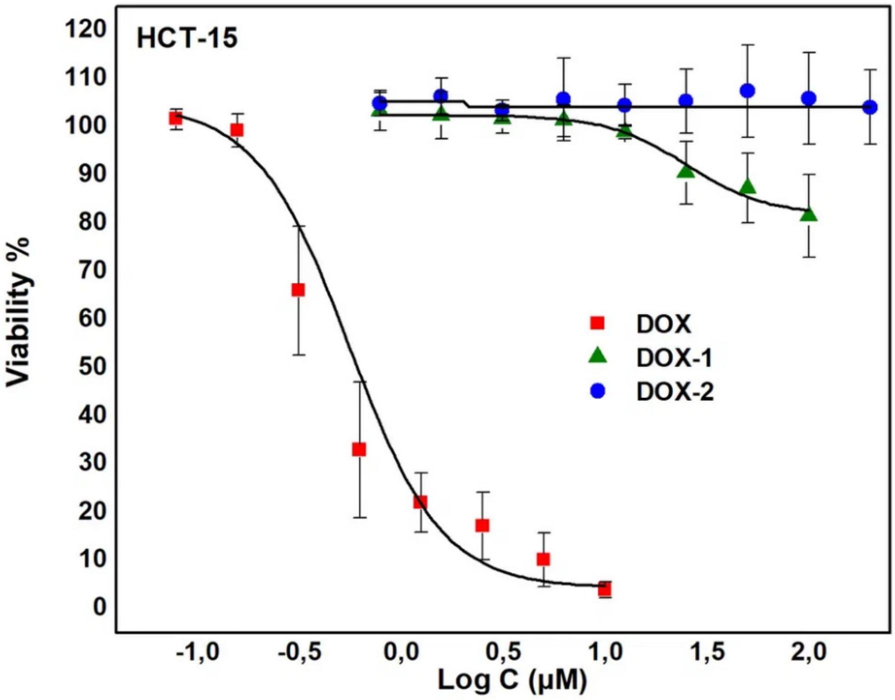
HCT-15
DOX
DOX-1
DOX-2
Viability %
Log C (µM)

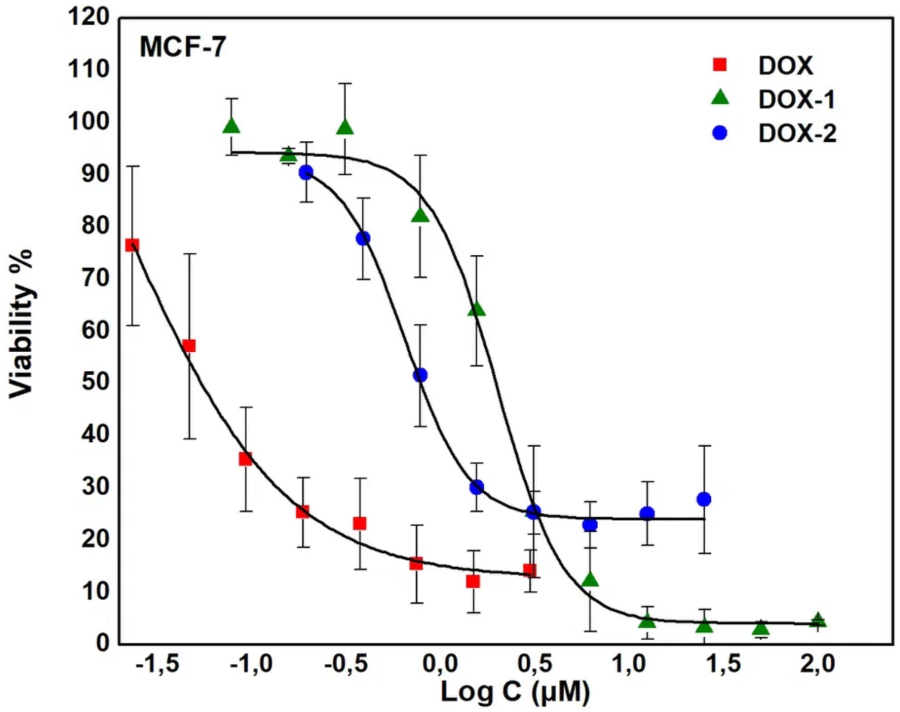
MCF-7
DOX
DOX-1
DOX-2
Viability %
Log C (µM)
120
110
100
90
80
70
60
50
40
30
20
10
0
-1,5
-1,0
-0,5
0,0
0,5
1,0
1,5
2,0

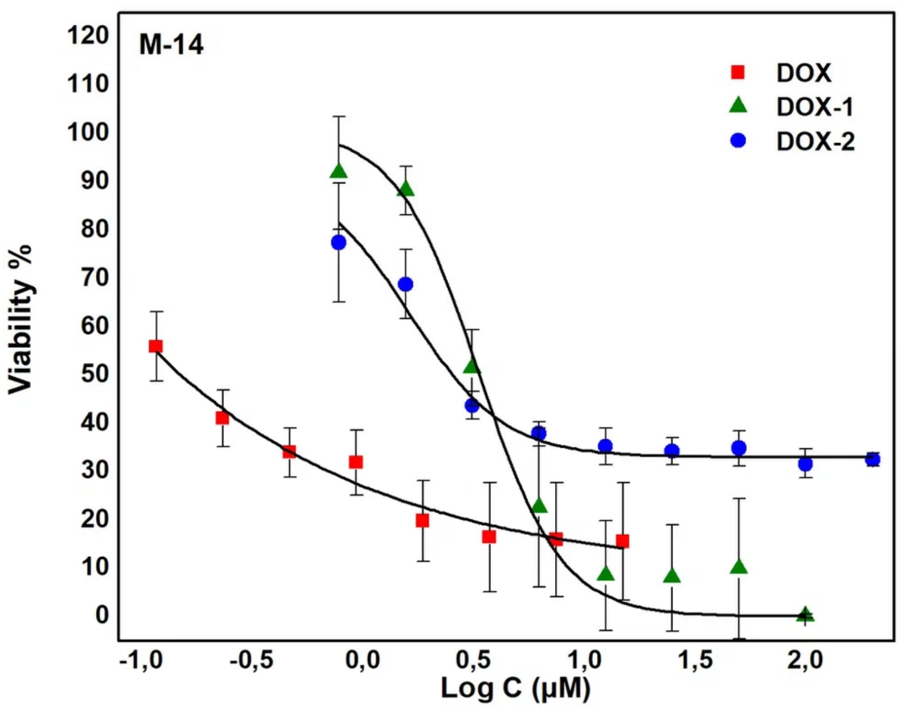
M-14
DOX
DOX-1
DOX-2
Viability %
Log C (µM)
120
110
100
90
80
70
60
50
40
30
20
10
0
-1,0
-0,5
0,0
0,5
1,0
1,5
2,0

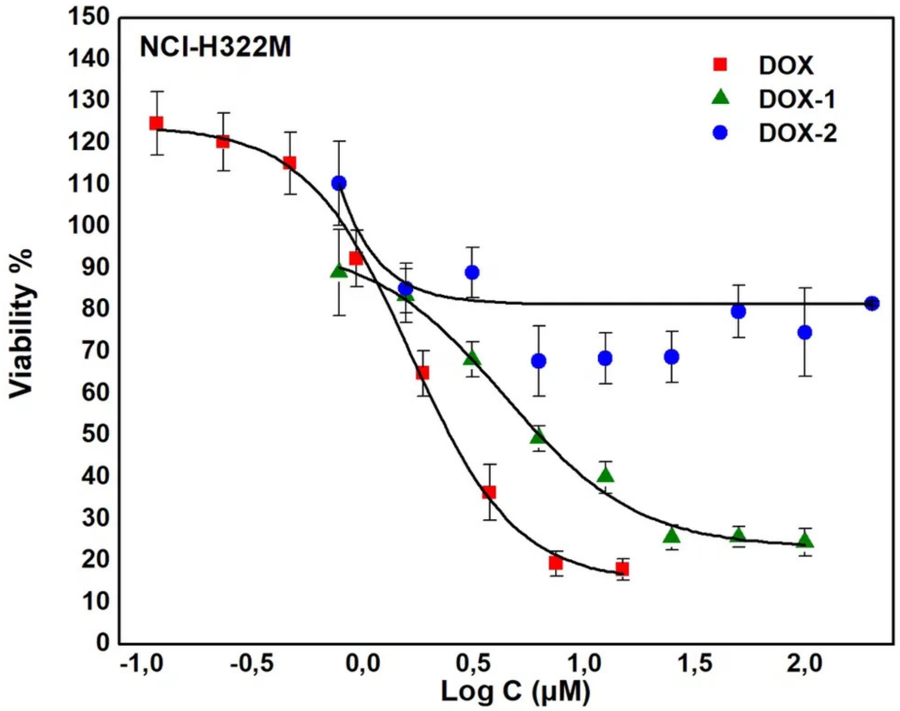
NCI-H322M
DOX
DOX-1
DOX-2
Viability %
Log C (μM)
150
140
130
120
110
100
90
80
70
60
50
40
30
20
10
0
-1,0
-0,5
0,0
0,5
1,0
1,5
2,0

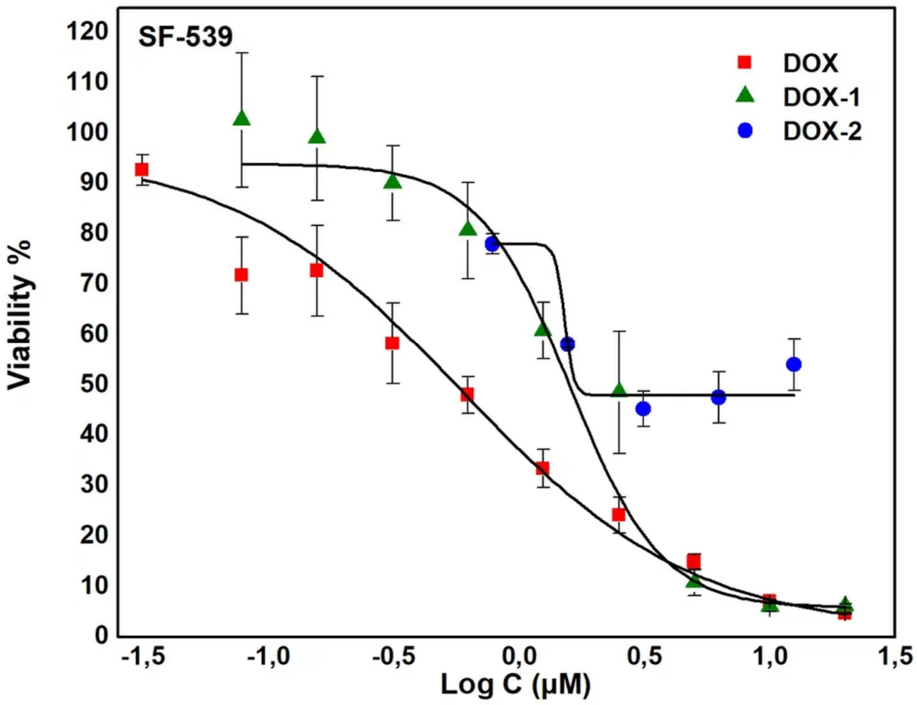
SF-539
DOX
DOX-1
DOX-2
Viability %
Log C (μM)
120
110
100
90
80
70
60
50
40
30
20
10
0
-1,5
-1,0
-0,5
0,0
0,5
1,0
1,5

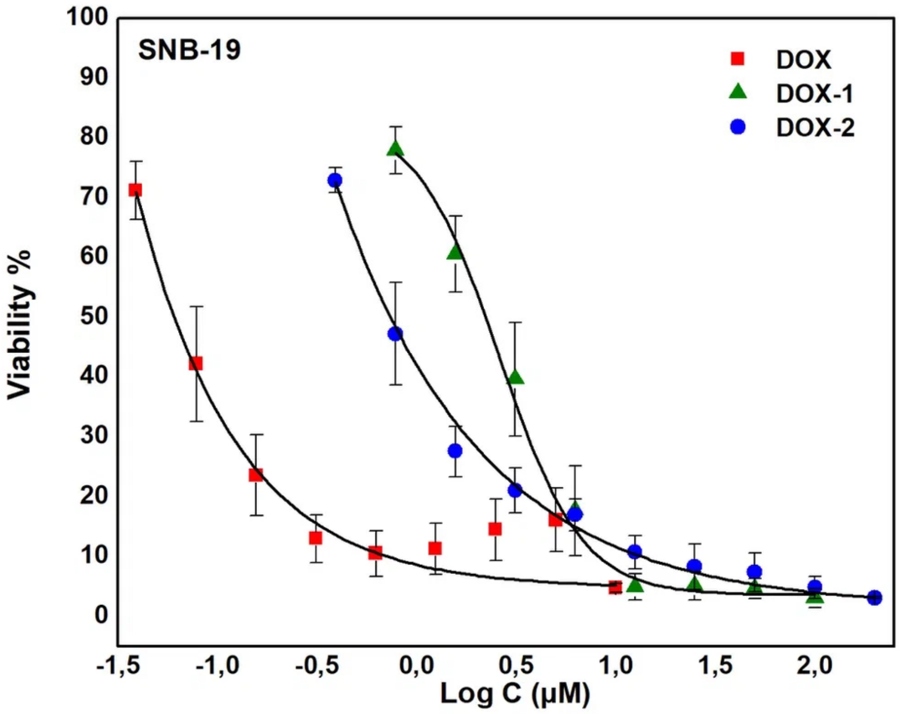
SNB-19
DOX
DOX-1
DOX-2
Viability %
Log C (μM)
100
90
80
70
60
50
40
30
20
10
0
-1,5
-1,0
-0,5
0,0
0,5
1,0
1,5
2,0

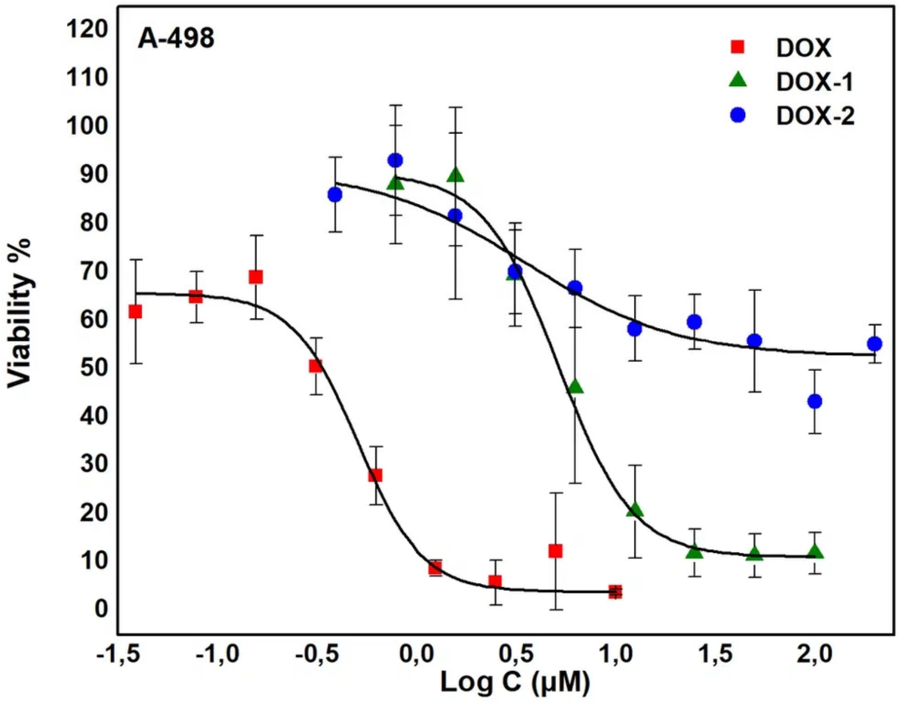
A-498
DOX
DOX-1
DOX-2
Viability %
Log C (μM)
120
110
100
90
80
70
60
50
40
30
20
10
0
-1,5
-1,0
-0,5
0,0
0,5
1,0
1,5
2,0

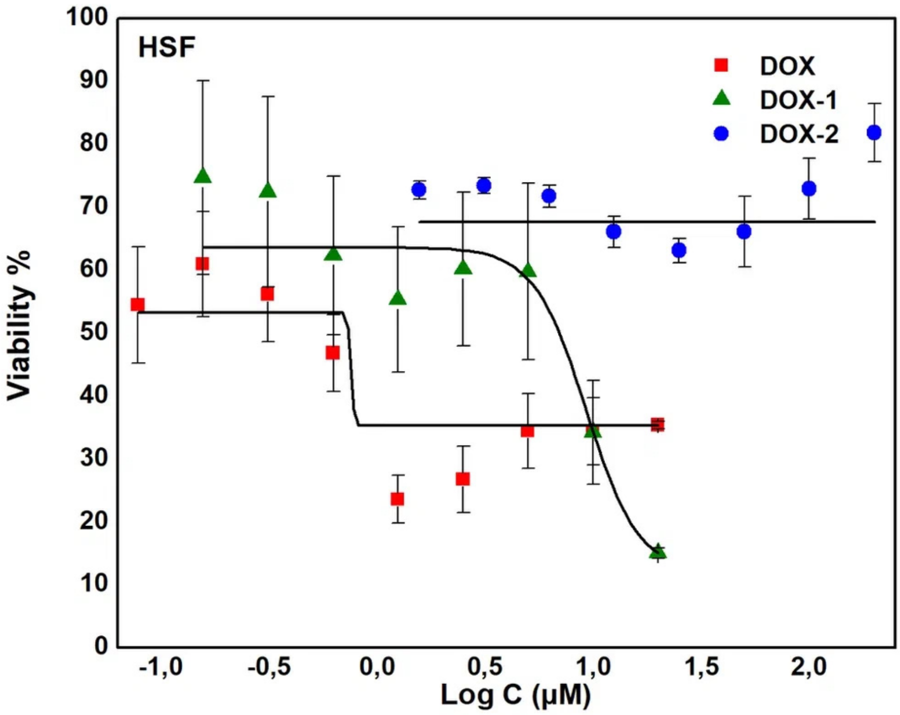
HSF
DOX
DOX-1
DOX-2
Viability %
Log C (μM)
100
90
80
70
60
50
40
30
20
10
0
-1,0
-0,5
0,0
0,5
1,0
1,5
2,0

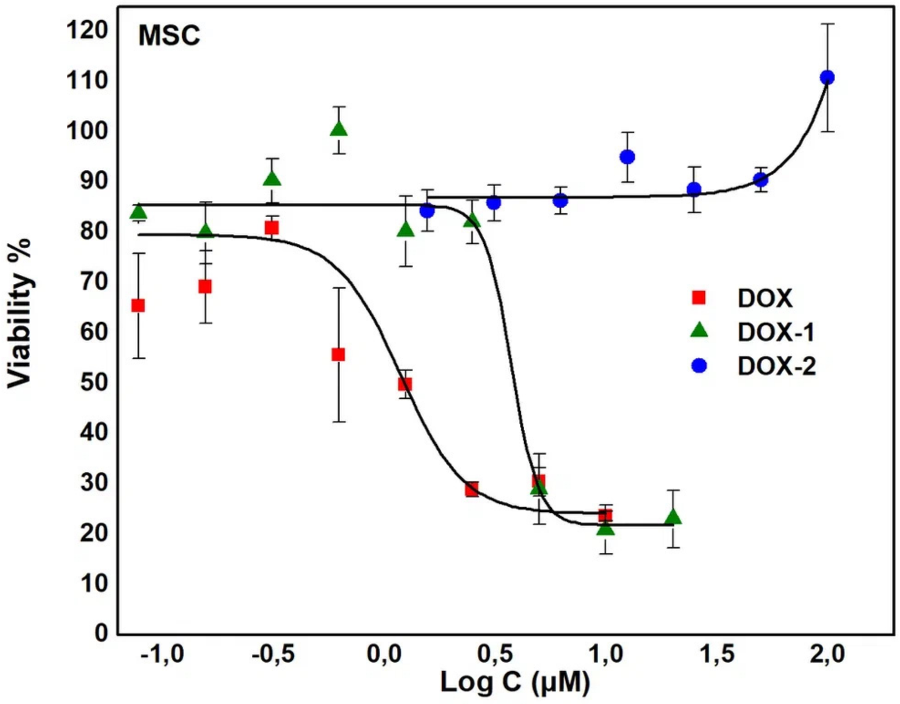
MSC
DOX
DOX-1
DOX-2
Viability %
Log C (μM)
120
110
100
90
80
70
60
50
40
30
20
10
0
-1,0
-0,5
0,0
0,5
1,0
1,5
2,0

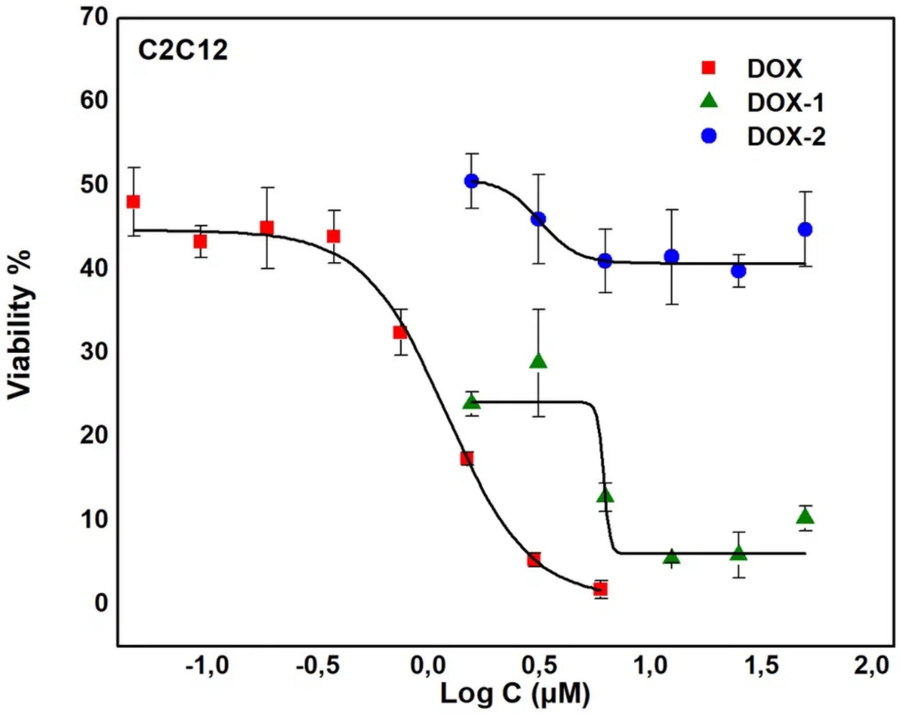

Figure S2. Appendix to Table 2. Sigmoidal dose-response relationships used to determine IC50 values of the studied compounds in the Colony formation assay. Data are averages of 2-3 independent repeats, mean ± SE.

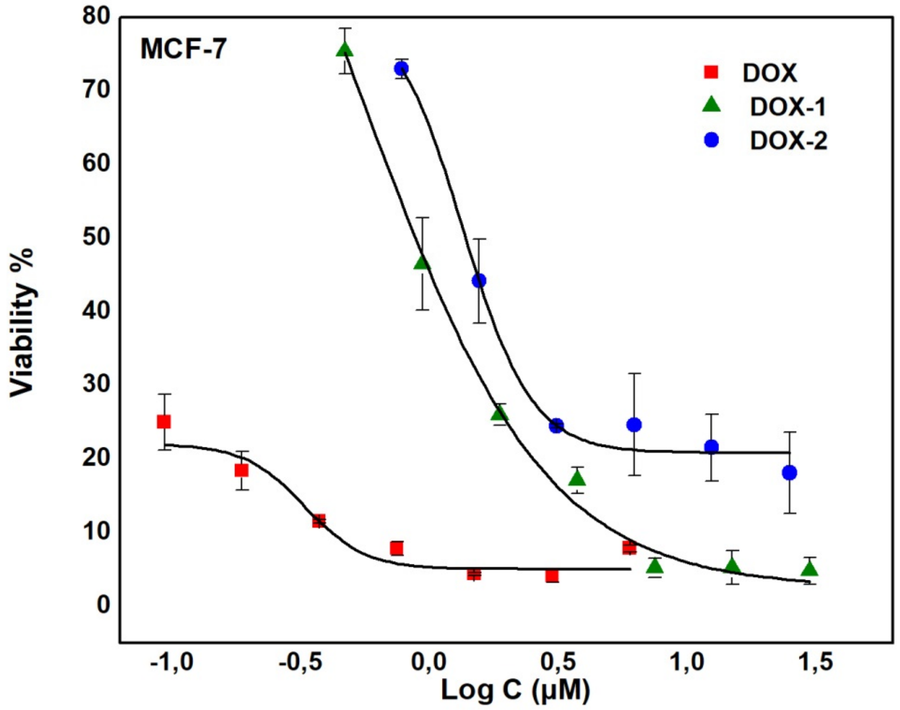

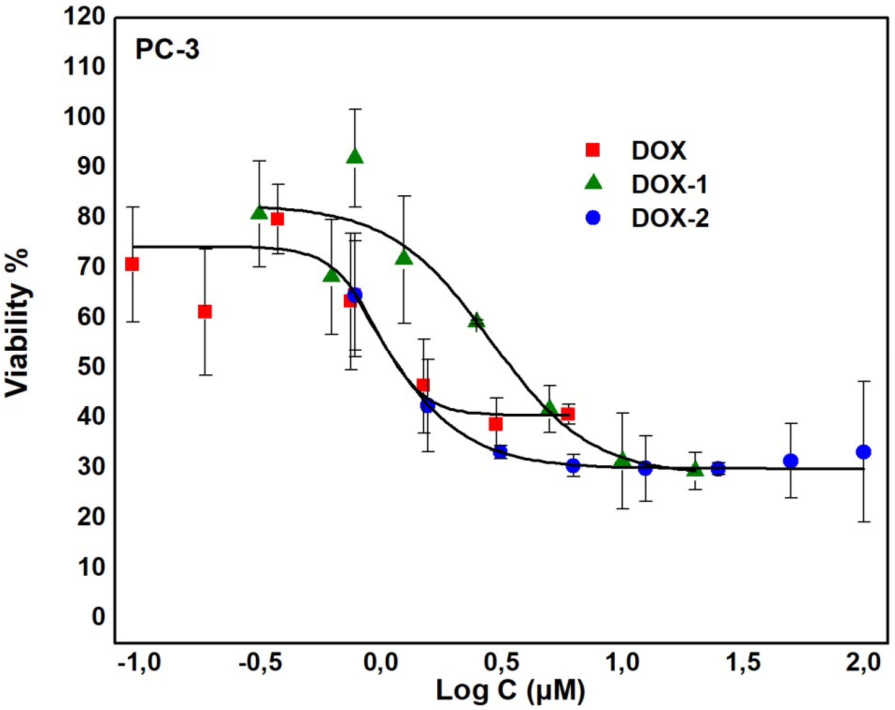
PC-3
DOX
DOX-1
DOX-2
Viability %
Log C (μM)

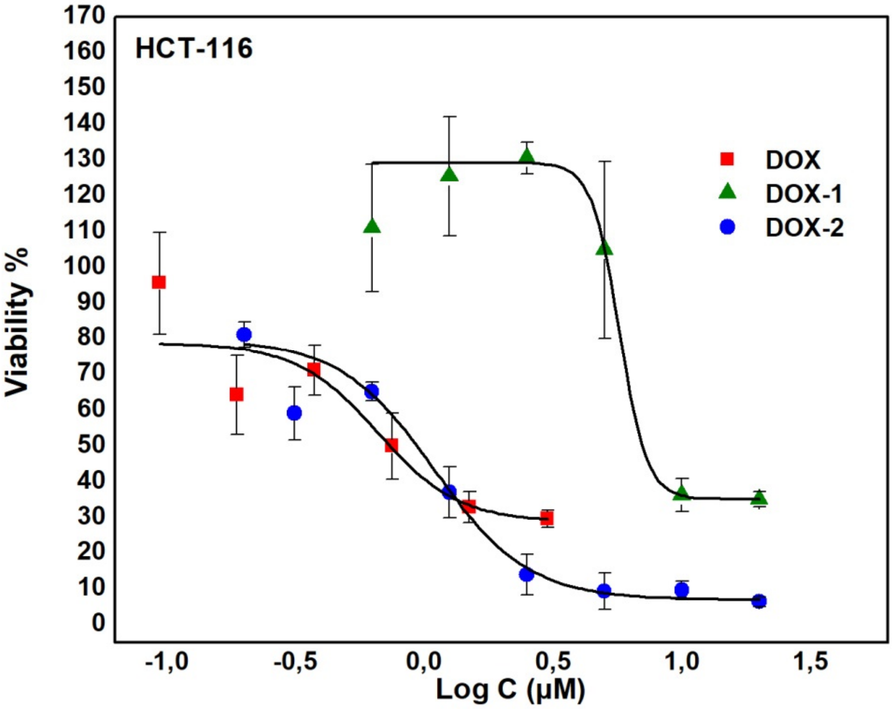
HCT-116
DOX
DOX-1
DOX-2
Viability %
Log C (μM)

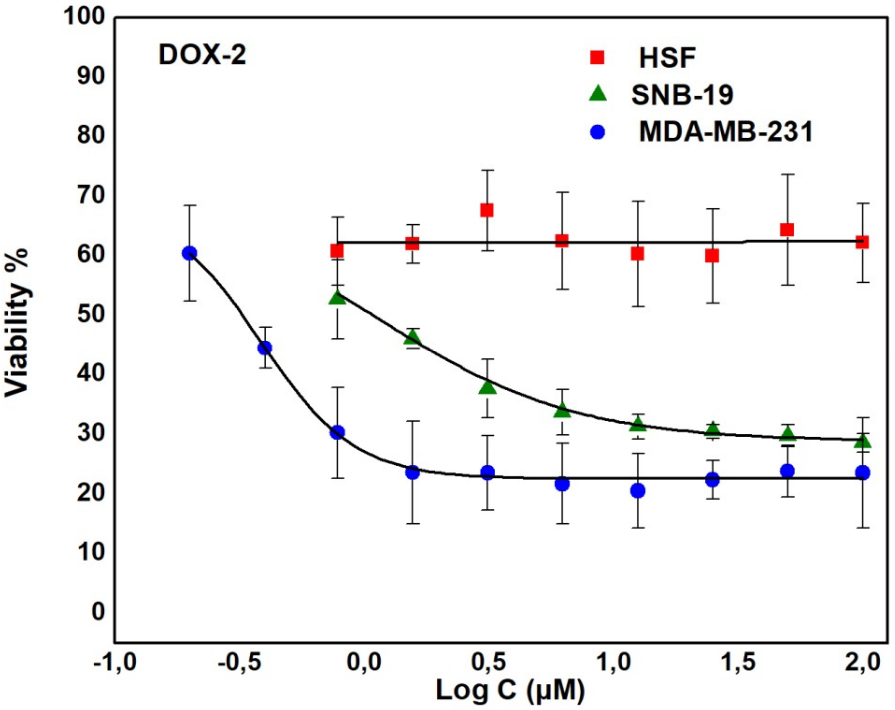
DOX-2
HSF
SNB-19
MDA-MB-231
Viability %
Log C (µM)
100
90
80
70
60
50
40
30
20
10
0
-1,0
-0,5
0,0
0,5
1,0
1,5
2,0

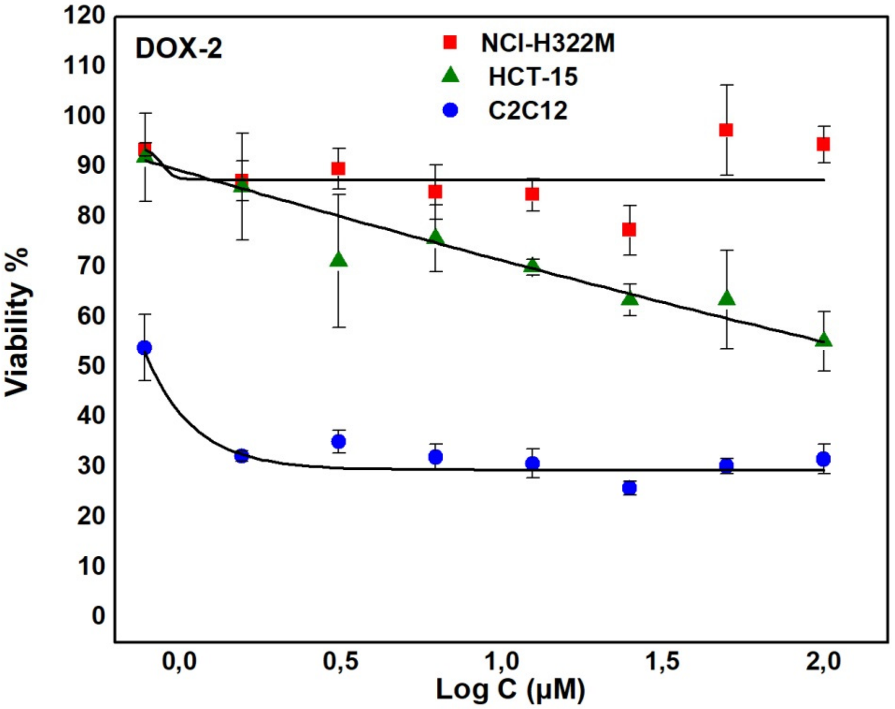
DOX-2
NCI-H322M
HCT-15
C2C12
Viability %
Log C (µM)
120
110
100
90
80
70
60
50
40
30
20
10
0
0,0
0,5
1,0
1,5
2,0
